# Low CD4 + T cell count is related to specific anti-nuclear antibodies, IFNα protein positivity and disease activity in systemic lupus erythematosus pregnancy

**DOI:** 10.1186/s13075-024-03301-0

**Published:** 2024-03-09

**Authors:** Agnes Torell, Marit Stockfelt, Kaj Blennow, Henrik Zetterberg, Tansim Akhter, Dag Leonard, Lars Rönnblom, Sofia Pihl, Muna Saleh, Christopher Sjöwall, Helena Strevens, Andreas Jönsen, Anders A. Bengtsson, Estelle Trysberg, Maria Majczuk Sennström, Agneta Zickert, Elisabet Svenungsson, Iva Gunnarsson, Johan Bylund, Bo Jacobsson, Anna Rudin, Anna-Carin Lundell

**Affiliations:** 1 Department of Rheumatology and Inflammation Research, Institute of Medicine, Sahlgrenska Academy at the University of Gothenburg, Guldhedsgatan 10A, 405 30 Gothenburg, Sweden; 2 Rheumatology, Sahlgrenska University Hospital, Gothenburg, Sweden; 3 Department of Psychiatry and Neurochemistry, Institute of Neuroscience and Physiology, Sahlgrenska Academy at the University of Gothenburg, Mölndal, Sweden; 4 Clinical Neurochemistry Laboratory, Sahlgrenska University Hospital, Mölndal, Sweden; 5 Paris Brain Institute, ICM, Pitié-Salpêtrière Hospital, Sorbonne University, Paris, France; 6 Neurodegenerative Disorder Research Center, Division of Life Sciences and Medicine and Department of Neurology, Institute On Aging and Brain Disorders, University of Science and Technology of China and First Affiliated Hospital of USTC, Hefei, People’s Republic of China; 7 Department of Neurodegenerative Disease, UCL Institute of Neurology, Queen Square, London, UK; 8 UK Dementia Research Institute at UCL, London, UK; 9 Hong Kong Center for Neurodegenerative Diseases, Clear Water Bay, Hong Kong, China; 10 Winsconsin Alzheimer’s Disease Research Center, School of Medicine and Public Health, University of Wisconsin, University of Wisconsin-Madison, Madison, WI USA; 11 Department of Women’s and Children’s Health, Section of Obstetrics and Gynecology, Uppsala University, Uppsala, Sweden; 12 Department of Medical Sciences, Rheumatology, Uppsala University, Uppsala, Sweden; 13 Department of Obstetrics and Gynecology, Linköping University Hospital, Linköping, Sweden; 14 Department of Biomedical and Clinical Sciences, Division of Children’s and Women’s Health, Linköping University, Linköping, Sweden; 15 Division of Inflammation and Infection, Department of Biomedical and Clinical Sciences, Linköping University, Linköping, Sweden; 16 Department of Obstetrics and Gynecology, Institute of Clinical Sciences, Skåne University Hospital, Lund, Sweden; 17 Department of Clinical Sciences Lund, Rheumatology, Lund University, Skåne University Hospital, Lund, Sweden; 18 Department of Womens and Childrens Health, Division for Obstetrics and Gynecology, Karolinska University Hospital, Karolinska Institute, Stockholm, Sweden; 19 Division of Rheumatology, Department of Medicine Solna, Karolinska Institute, Karolinska University Hospital, Stockholm, Sweden; 20 Department of Oral Microbiology and Immunology, Institute of Odontology, Sahlgrenska Academy at the University of Gothenburg, Gothenburg, Sweden; 21 Department of Obstetrics and Gynecology, Sahlgrenska University Hospital, Gothenburg, Sweden; 22 Department of Obstetrics and Gynecology, Sahlgrenska Academy at the University of Gothenburg, Gothenburg, Sweden; 23 Department of Genetics and Bioinformatics, Division of Health Data and Digitalisation, Institute of Public Health, Oslo, Norway

**Keywords:** Systemic lupus erythematosus (SLE), Lymphocyte count, Pregnancy, Interferon alpha, Autoantibodies

## Abstract

**Background:**

Lymphopenia, autoantibodies and activation of the type I interferon (IFN) system are common features in systemic lupus erythematosus (SLE). We speculate whether lymphocyte subset counts are affected by pregnancy and if they relate to autoantibody profiles and/or IFNα protein in SLE pregnancy.

**Methods:**

Repeated blood samples were collected during pregnancy from 80 women with SLE and 51 healthy controls (HC). Late postpartum samples were obtained from 19 of the women with SLE. Counts of CD4 + and CD8 + T cells, B cells and NK cells were measured by flow cytometry. Positivity for anti-nuclear antibodies (ANA) fine specificities (double-stranded DNA [dsDNA], Smith [Sm], ribonucleoprotein [RNP], chromatin, Sjögren’s syndrome antigen A [SSA] and B [SSB]) and anti-phospholipid antibodies (cardiolipin [CL] and β_2_ glycoprotein I [β_2_GPI]) was assessed with multiplexed bead assay. IFNα protein concentration was quantified with Single molecule array (Simoa) immune assay. Clinical data were retrieved from medical records.

**Results:**

Women with SLE had lower counts of all lymphocyte subsets compared to HC throughout pregnancy, but counts did not differ during pregnancy compared to postpartum. Principal component analysis revealed that low lymphocyte subset counts differentially related to autoantibody profiles, cluster one (anti-dsDNA/anti-Sm/anti-RNP/anti-Sm/RNP/anti-chromatin), cluster two (anti-SSA/anti-SSB) and cluster three (anti-CL/anti-β2GPI), IFNα protein levels and disease activity. CD4 + T cell counts were lower in women positive to all ANA fine specificities in cluster one compared to those who were negative, and B cell numbers were lower in women positive for anti-dsDNA and anti-Sm compared to negative women. Moreover, CD4 + T cell and B cell counts were lower in women with moderate/high compared to no/low disease activity, and CD4 + T cell count was lower in IFNα protein positive relative to negative women. Finally, CD4 + T cell count was unrelated to treatment.

**Conclusion:**

Lymphocyte subset counts are lower in SLE compared to healthy pregnancies, which seems to be a feature of the disease per se and not affected by pregnancy. Our results also indicate that low lymphocyte subset counts relate differentially to autoantibody profiles, IFNα protein levels and disease activity, which could be due to divergent disease pathways.

**Supplementary Information:**

The online version contains supplementary material available at 10.1186/s13075-024-03301-0.

## Background

Systemic lupus erythematosus (SLE) is an autoimmune disease that afflicts mostly women, often in fertile ages [1]. An aberrant activation in many aspects of the immune system has been documented for SLE, but common denominators for most patients include T cell-dependent autoantibody production and type I interferon (IFN) overexpression [2–4]. The widely distributed immune activation is reflected by a diversity in laboratory abnormalities (including lymphopenia, hypocomplementemia and presence of autoantibodies) and in clinical features (including arthritis, renal and dermatological manifestations) [4]. Moreover, SLE pregnancy carries a risk for disease flare and an increased risk of pregnancy complications compared to the general population [5, 6].

Lymphopenia is common in individuals with SLE, occurring in about 40% of the patients [7–9], and low absolute numbers of the lymphocyte subsets CD4 + T cells, CD8 + T cells, B cells and NK cells have been reported in patients with SLE compared to healthy individuals [10–12]. In SLE, lymphopenia is independently associated with organ damage accrual, neurological involvement and disease activity [8, 9, 13], but it is unknown whether specific lymphocyte subset numbers in blood are affected by pregnancy in SLE and if subset counts relate to disease activity during pregnancy.

Various autoantibodies are described in SLE [14], but only a few are assessed routinely in the clinical setting. These include anti-nuclear antibodies (ANA) directed to double stranded DNA (dsDNA), Smith antigen (Sm), ribonucleoprotein (RNP), Sjögren’s syndrome antigen A (SSA) and B (SSB) and anti-phospholipid antibodies (aPL) directed to cardiolipin (CL) and β2-glycoprotein I (β_2_GPI) [15]. The heterogeneity of SLE has motivated attempts to stratify patients into subgroups based on disease-related autoantibody profiles in non-pregnant patients with SLE [16–18]. A large international longitudinal study recently identified four serological clusters that differed in clinical features but also predicted long term events [16]. The latter and other studies also report that positivity for most SLE-related autoantibodies decrease over time [16, 19, 20]. In cross-sectional setting, one study separated SLE patients into three groups where two were dominated by anti-dsDNA-positive individuals who had a higher frequency of lymphopenia compared to the third group that included fewer anti-dsDNA-positive individuals [18]. Another study revealed four groups where the first was dominated by anti-SSA/SSB positivity, the second by anti-Sm/dsDNA/RNP positivity, the third by aPL positivity and the last was seronegative [17]. Lymphopenia was more frequent and disease activity higher in the seropositive groups compared to the seronegative group [17]. It is still unknown if counts of specific lymphocyte subsets including T cells, B cells and NK cells relate to different disease-related autoantibody profiles, and if they relate to each other during pregnancy in SLE.

Many patients with SLE present with increased expression of type I interferon (IFN)-regulated genes in blood cells and in tissue, an IFN signature [2, 3], and a single cell RNA sequencing analysis of PBMC showed that increased expression of type I IFN regulated genes in monocytes correlate with low naïve CD4 + T cells in SLE [21]. Individuals with SLE also have higher IFNα protein concentrations in blood compared to healthy subjects as demonstrated by the use of an ultrasensitive single molecule array (Simoa) digital enzyme-linked immunosorbent assay (ELISA) [22]. In SLE, Simoa-quantified IFNα protein levels strongly correlate with a whole blood IFN-I gene score, and these methods identify associtations with SLE disease activity equally well [23]. Using the digital ELISA technology, we reported that IFNα protein positivity is present in a subgroup of pregnant women with SLE, but the protein concentrations are similar during pregnancy and in the late postpartum period [24]. Additionally, we and others have reported that pregnant and non-pregnant SLE patients positive for anti-SSA antibodies have increased IFNα protein levels in blood [24, 25]. Yet, it is unknown if lymphocyte subset counts relate to IFNα protein levels in pregnant women with SLE.

The first aim of this study was to compare total CD4 + T cell, CD8 + T cell, B cell and NK cell counts prospectively throughout pregnancy in women with SLE relative to the late postpartum period and to healthy pregnant women. Secondly, we aimed to investigate whether the lymphocyte subset counts were related to autoantibody profiles, IFNα protein levels, disease activity and gestational age at birth in SLE pregnancy.

## Materials And Methods

### Cohort

This Swedish multicenter study enrolled pregnant women with SLE (*n* = 80) meeting the 1997 American College of Rheumatology (ACR) and/or the 2012 Systemic Lupus International Collaborating Clinics (SLICC) classification criteria [26, 27] between November 2018 and June 2022 at Rheumatology clinics in: Gothenburg (Sahlgrenska University hospital, *n* = 24), Stockholm (Karolinska University Hospital, *n* = 38), Uppsala (Uppsala University Hospital, *n* = 3), Linköping (Linköping University Hospital, *n* = 6) and Lund (Skåne University Hospital, *n* = 9). Healthy pregnant women (HC, *n* = 51) were enrolled at one antenatal clinic in Gothenburg (Regionhälsan, Gothenburg) between October 2018 and December 2022. Most pregnant women with SLE and HC were included at 10–12 weeks of gestation and followed in the second (week 18–20) and third (week 32–34) trimester. Disease activity was evaluated according to the SLE Disease Activity Index 2000 (SLEDAI-2 K) [28], and measured at least once between week 10 and week 34 and if the disease activity was assessed more than once, the highest score was used in analyses. The number of pregnant women with SLE for whom SLEDAI-2 K assessments were obtained from in each trimester is shown in Supplementary Table 1. Clinical data including disease duration, medication, ever autoantibody positivity, gestational age at birth and giving birth to a small for gestational age (SGA) infant were retrieved from medical records. SGA (*n* = 13) was defined as birth weight less than the 10th percentile for expected birth weight [29]. Exclusion criteria were inability to understand the study-related patient information and informed consent form, presence of other serious disease, including active cancer and other rheumatic autoimmune diseases, or treatment with anti-BAFF or anti-CD20 antibodies within 12 months before inclusion. Women who had miscarriage during trimester one were excluded. None of the women with SLE were treated with anifrolumab before or during their pregnancy. All participants have given their written informed consent and the Ethics board in Gothenburg (Dnr 404–18) and The Swedish Ethical Review Authority (amendments Dnr 2020–05101 and Dnr 2022–01158-02) have approved the study.

### Sample collection

Peripheral blood samples were collected in heparinized tubes from pregnant women with SLE and HC in the first, second and third trimester. One additional blood sample was collected late postpartum from 19 of the women with SLE (at least six months after delivery (median 10 [6,–36] months). Information about the number of blood samples collected for each trimester is presented in Supplementary Table 2. All blood samples were kept at ambient temperature until processed the day after, within 24 h after venipuncture at our laboratory in Gothenburg. Whole blood was used for flow cytometry analysis of total lymphocyte subset counts. Density centrifugation of whole blood was performed to isolate plasma that was kept frozen (-80 °C) until further analysis.

### Autoantibody status during pregnancy in SLE

Analysis of positivity for immunofluorescence (IF)-ANA, for ANA fine specificities including antibodies to dsDNA, Sm, RNP, SSA, SSB, chromatin and ribosomal P protein, and for anti-phospholipid antibodies including anti-CL and anti-β_2_GPI in plasma collected during pregnancy was performed at the accredited laboratory of Clinical Immunology, Sahlgrenska University Hospital. Most of these samples were collected in trimester three and plasma was diluted 1:1 in PBS. IF-ANA was analyzed using Hep-2 cells according to routine, and 66 out of 80 (83%) women with SLE were positive. ANA fine specificities were analyzed using multiplexed bead technology by the BioPlex™ 2200 System (BioRad Laboratories, Hercules, USA). The cut-off for most ANA-specificities was 1.0 AI (antibody index) except for anti-chromatin (1.5 AI), anti-RNP (3.0 AI) and anti-dsDNA (10 IU/mL). Positive ANA-specificities were confirmed with another method according to the manufacturers recommendation: *Crithidia luciliae* test for anti-dsDNA (ImmunoConcept, Sacramento, CA), automated ELISA-based test system Alegria® (Orgentec Diagnostics, Mainz, Germany) for anti-SSA52 and line blot ANA Profile 5 IgG for all other ANA-specificities (Euroimmun, Lübeck, Germany). Anti-CL and anti-β_2_GPI of IgG isotype were examined using the BioPlex™ 2200 multiplex immunoassay system and APLS reagents. Cut-off values for positivity were 20 GPL for aCL IgG and 20 AU/mL for anti-β2GPI IgG as recommended by the manufacturer. Ever autoantibody status was obtained from medical records and included positivity for anti-dsDNA/Sm/SSA/SSB, anti-CL/β_2_GPI and lupus anticoagulant (LAC). The method for analysis of ever antibody positivity differed between the study sites and dsDNA was confirmed by either *Crithidia luciliae* test or ELISA. Positivity was determined according to cut-off levels at the local laboratories.

### Flow cytometry

TruCount™ assay was used to analyze total number of lymphocytes, CD4 + T cells, CD8 + T cells, B cells and NK cells in whole blood. In brief, blood and antibodies against CD45, CD3, CD4, CD8, CD19, CD20 and CD56 (Supplementary Table 3) were added to BD TruCount™ tubes (BD Bioscience) and incubated for 15 min. Red blood cells were then lysed using BD FACS™ Lysing solution (BD Sciences). All samples were acquired in a FACSVerse equipped with FACSuite Software (BD Biosciences) and analyzed with FlowJo Software (TreeStar, Ashland, Oregon, USA).

### IFNα protein quantification

The concentration of IFNα protein levels in plasma diluted 1:1 in PBS was quantified with Single molecule array (Simoa) digital ELISA on a HD-X Analyzer (Quanterix, Billerica, MA). To prevent false positive results the Simoa assay contained an inhibitor for heterophilic antibodies. If the concentration in a sample was below the lower limit of quantification (70 fg/ml) its value was adjusted to 35 fg/ml. IFNα positivity was defined as protein levels ≥ 136 fg/ml, representing three standard deviations above mean IFNα protein concentration among healthy blood donors [30].

### Statistical analysis

Multivariate data analysis was performed using the SIMCA-P software (Sartorius Stedem Biotech, Goettingen, Germany). Principal Component Analysis (PCA) was used to obtain an unsupervised descriptive overview of groupings and trends, associations, between total number of CD4 + T cells, CD8 + T cells, B cells and NK cells, IFNα protein levels, autoantibody positivity during pregnancy, disease activity, gestational age at birth and SGA among pregnant women with SLE. Orthogonal partial least squares (OPLS) analysis was performed to investigate medication or gestational age at birth (Y-variables) in relation to total numbers of T cells, B cells and NK cells (X-variables) in pregnant women with SLE. In the PCA and OPLS models default settings were used; data were centered and scaled to unit-variance to give all variables equal weight. Model quality was based on R2 and Q2 parameters that are presented in each figure. Univariate analyses were only performed for the strongest associations found in the PCA and OPLS models and included Kruskal–Wallis followed by Dunn’s multiple comparison test, Mann–Whitney U test and Spearman rank correlation test (GraphPad prism software, La Jolla, CA, USA) as described in each respective figure legend. *P*-values of < 0.05 were considered statistically significant.

## Results

### Clinical characteristics of pregnant women with SLE

Demographic and clinical data of the cohort are shown in Table 1. The data presented, except for the cross-sectional analysis of autoantibody positivity, have been previously described for subsets of both patients and controls [24]. In brief, the age and percentage of nulliparous women were similar in SLE and HC. For pregnant women with SLE, the median disease activity according to SLEDAI-2 K was 2 during pregnancy. Disease activity did not vary much between the trimesters (Supplementary Fig. 1A-B). For all patients with moderate/high disease activity (SLEDAI-2 K ≥ 4), the components that contributed to the score were due to the disease per se and not to pregnancy, and the most common features were increase in anti-dsDNA, low complement, and arthritis. Historically, all except one were ever ANA-positive by immunofluorescence and the majority were ever anti-dsDNA positive. In the cross-sectional analysis of autoantibody positivity during pregnancy, 71% were positive for at least one of the ANA fine specificities assessed. In line with previous findings [19, 20], the percentage of positive women was mostly lower in cross-sectional compared to ever positive analysis: anti-dsDNA (36% vs. 84%), anti-Sm (15% vs 25%), anti-SSB (10% vs 14%), anti-CL (8% vs 16%) and anti-β2GPI (9% vs 20%). Moreover, 30% were positive for anti-chromatin, 23% for anti-Sm/RNP, 14% for anti-RNP, and 3% for anti-Ribosomal P during pregnancy. Most women with SLE were treated with hydroxychloroquine (93%) and acetylsalicylic acid (88%) during pregnancy, while one third or less were treated with prednisone (33%), azathioprine (29%) and/or low molecular weight heparin (24%).

**Table 1:** Characteristics of women with SLE and healthy controls

	SLE (*n* = 80)		Controls (*n* = 51)
Age (years), median (range)^a^	32 (23–43)		32 (18–41)
Nulliparous, n (%)	48 (60)		35 (69)
Disease duration at inclusion (years), median (range)	9 (0–26)		
SLEDAI-2 K during pregnancy, median (range)^b^	2 (0–18)		
ACR criteria, n (%)	ever		
Malar rash	31 (39)		
Discoid rash	6 (8)		
Photosensitivity	39 (49)		
Oral ulcers	30 (38)		
Arthritis	66 (83)		
Serositis	17 (21)		
Renal disorder	30 (38)		
Neurological disorder	5 (6)		
Hematological disorder	53 (66)		
Immunological disorder	69 (86)		
Anti-nuclear antibody^c^	79 (99)		
Antiphospholipid syndrome, n (%)	5 (6)		
Autoantibodies, n (%)	ever	during pregnancy	
*ANA fine specificity*			
Any ANA fine specificity	N/A	57 (71)	
Anti-dsDNA	67 (84)	28 (36)	
Anti-Sm	20 (25)^b^	12 (15)	
Anti-SSA	23 (29)	24 (30)	
Anti-SSB	11 (14)	8 (10)	
Anti-Sm/RNP	N/A	18 (23)	
Anti-RNP	N/A	11 (14)	
Anti-Chromatin	N/A	24 (30)	
Anti-Ribosomal P	N/A	2 (3)	
*Antiphospholipid antibodies*			
Anti-cardiolipin IgG	13 (16)	6 (8)	
Anti-β2glycoprotein I IgG	16 (20)^b^	7 (9)	
Lupus Anticoagulant	12 (15)	N/A	
Medication, n (%)^d^			
Hydroxychloroquine or chloroquine phosphate		74 (93)	
Acetylsalicylic acid		70 (88)	
Low molecular weight heparin		19 (24)	
Azathioprine		23 (29)	
Prednisone		26 (33)	

^a^Age of mother at gestational week 12

^b^Missing data from one patient

^c^Positive by immunofluorescence microscopy (IF-ANA)

^d^Early pregnancy

### Blood lymphocyte subset counts are not affected by pregnancy in SLE but are lower compared to healthy controls

**Fig. 1:**
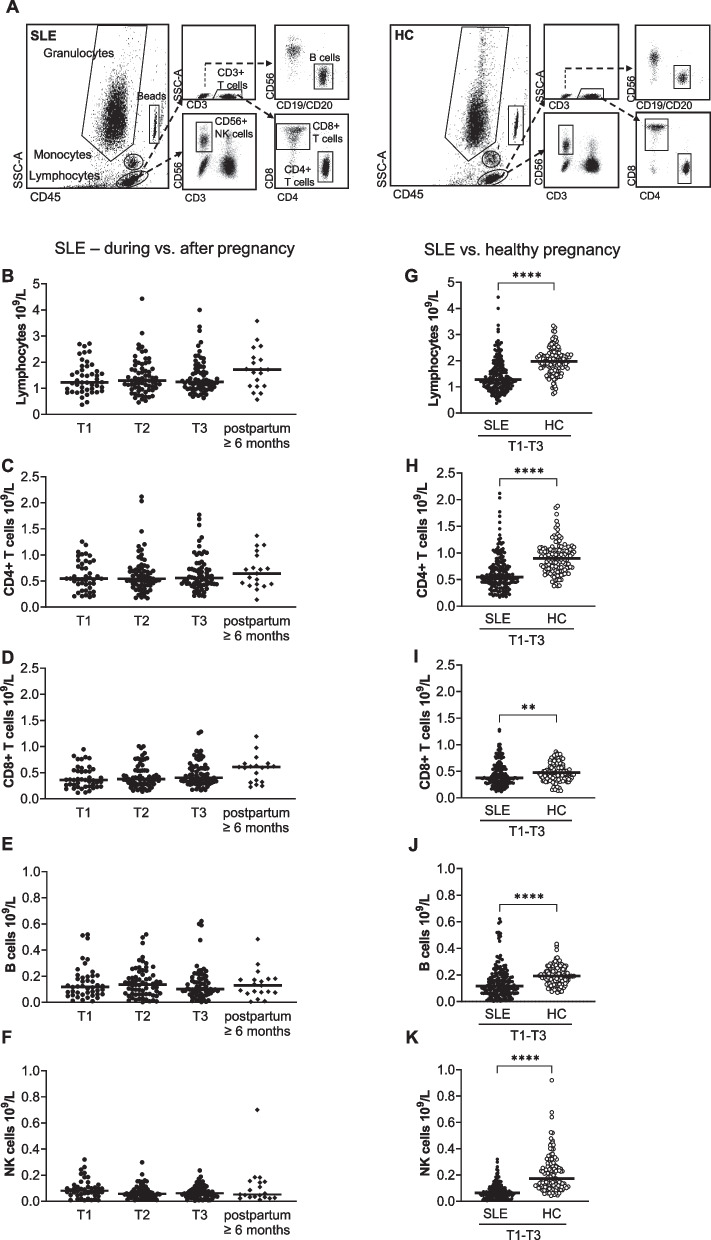
Blood lymphocyte subset counts are not affected by pregnancy in SLE. **(A)** Representative flow cytometry plots from SLE and HC illustrating the gating strategy for total number of lymphocytes, CD4 + T cells, CD8 + T cells, B cells and NK cells respectively. Total number of **(B)** lymphocytes, **(C)** CD4 + T cells, **(D)** CD8 + T cells, **(E)** B cells and **(F)** NK cells in trimester one, two, three and late postpartum in women with SLE. Combined data from trimester one, two and three comparing numbers of **(G)** total lymphocytes, **(H)** CD4 + T cells, **(I)** CD8 + T cells, **(J)** B cells and **(K)** NK cells between SLE and HC pregnancy. **p < 0.01, ****p < 0.0001, **(G-K)** Mann–Whitney U test

**Fig. 2:**
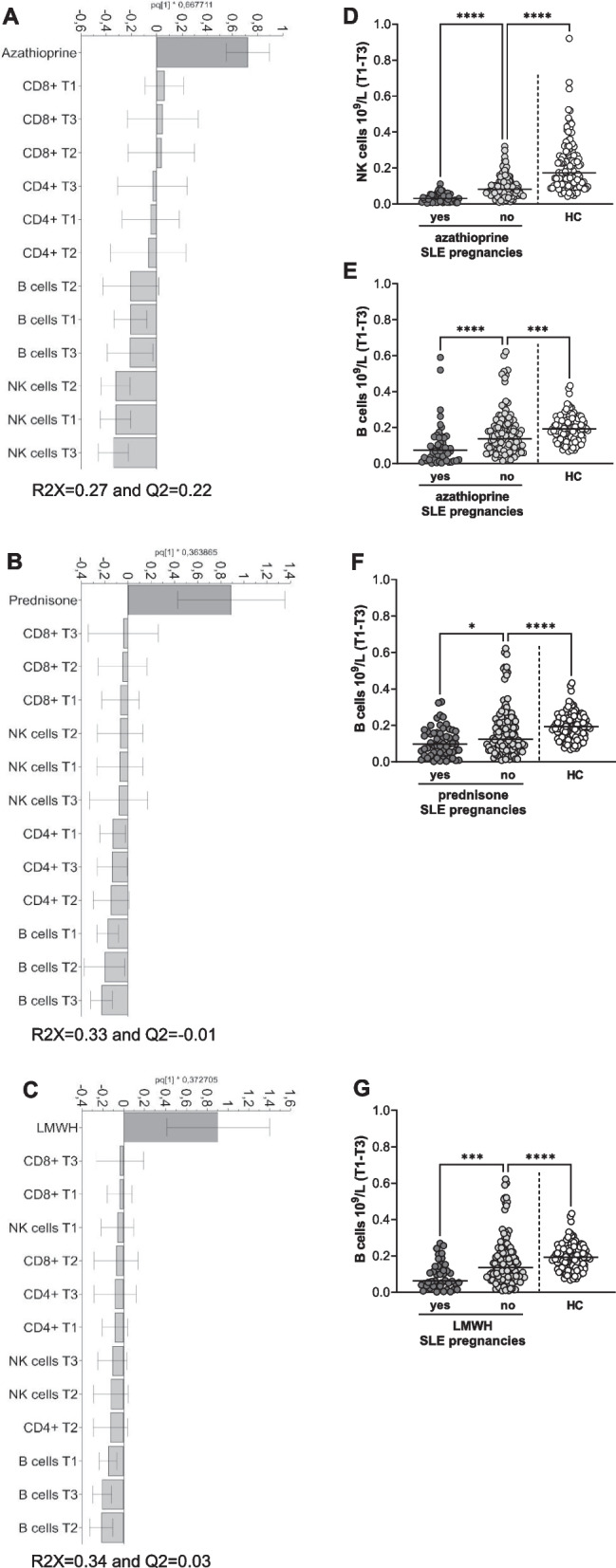
Treatment-independent decrease of NK cells and B cells in SLE pregnancies. OPLS loading column plots depicting counts of lymphocyte subsets positively or negatively associated with **(A)** azathioprine, **(B)** prednisone or **(C)** low molecular weight heparin. **(D)** NK cell and **(E)** B cell counts in patients treated or not with azathioprine and in HC. **(F)** B cell counts in patients treated or not with prednisone and in HC. **(G)** B cell counts in patients treated or not with low molecular weight heparin and in HC. *p < 0.05, ***p < 0.001, ****p < 0.0001, **(D-E)** Kruskal–Wallis followed by Dunn’s multiple comparison test

We first examined total numbers of circulating lymphocytes and the lymphocyte subsets CD4 + T cells, CD8 + T cells, B cells and NK cells in pregnant women with SLE and HC. A representative gating strategy for the different lymphocyte subsets in SLE and HC is presented in Fig. 1A. Late postpartum samples from pregnant women with SLE were analyzed to determine if potential differences in lymphocyte subset numbers were an effect of SLE combined with pregnancy or to SLE *per se*. Neither total lymphocyte count, nor the subsets CD4 + T cells, CD8 + T cells, B cells and NK cells differed significantly between trimesters or compared to late postpartum in SLE (Fig. 1B-F). Similar results were observed when only including data of women from whom late postpartum samples were collected (Supplementary Fig. 2A-E). However, total lymphocyte count and all subsets were significantly lower in SLE compared to HC throughout pregnancy (Fig. 1G-K, combined data from trimester one to three). The comparisons of cell counts between pregnant women with SLE and pregnant HC for each trimester separately are shown in Supplementary Fig. 3A-E. We also examined whether treatment related to lymphocyte subset counts in pregnant women with SLE. OPLS analysis indicated an inverse relation between azathioprine treatment and numbers of NK cells and B cells in all trimesters (Fig. 2A). Prednisone and low molecular weight heparin related inversely to B cell counts in all trimesters (Fig. 2B-C). These associations were corroborated in univariate analysis. NK cell and B cell counts were significantly lower in women treated compared to not treated with azathioprine (Fig. 2D-E). B cell counts were also lower in women treated compared to not treated with prednisone or heparin, respectively (Fig. 2F-G). Still, significantly lower numbers of both NK cells and B cells were found in women without respective treatment compared to HC (Fig. 2D-G). Treatment with hydroxychloroquine and acetylsalicylic acid were unrelated to lymphocyte subset counts (Supplementary Fig. 4A-B). In summary, pregnant women with SLE present with lower number of lymphocyte subset counts in blood relative to pregnant HC, which seem to be a feature of SLE *per se* and in part by treatment with immunosuppressive drugs and heparin but not related to pregnancy.

### Lymphocyte subset numbers inversely associate with positivity for ANA specificities, IFNα protein levels and disease activity in SLE pregnancy

**Fig. 3:**
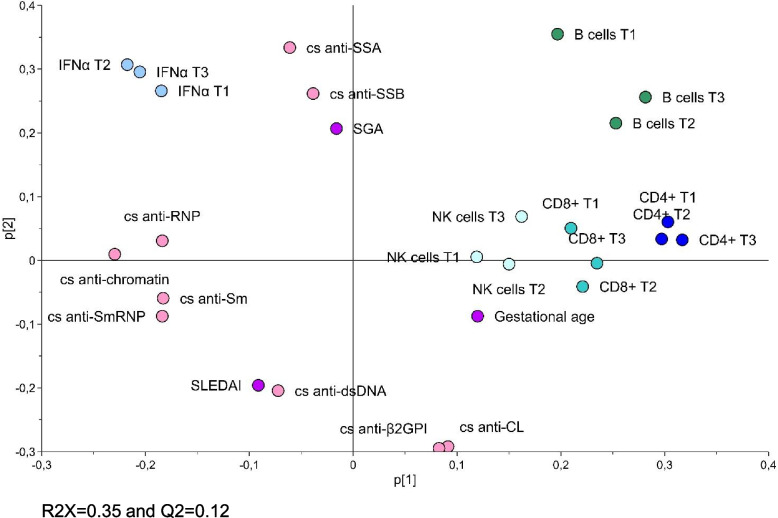
Lymphocyte subset counts inversely relate to autoantibody positivity, IFNα and disease activity in SLE pregnancy. Unsupervised principal component analysis demonstrating the relationship between number of CD4 + T cells, CD8 + T cells, B cells, NK cells in trimester one to three, cross sectional (cs) autoantibody positivity, IFNα protein levels, SLEDAI-2 K, gestational age at birth and small for gestational age (SGA) in pregnant women with SLE

We next performed PCA to investigate how CD4 + T cell, CD8 + T cell, B cell and NK cell counts relate to IFNα protein levels, autoantibody positivity during pregnancy, as well as to SLEDAI-2 K, gestational age at birth and SGA in SLE. We previously reported that plasma IFNα protein positivity is present in a subgroup (36%) of pregnant women with SLE [24]. As shown in Fig. 3, lymphocyte subset counts were projected on the opposite side to positivity for ANA specificities, IFNα protein levels and SLEDAI-2 K indicating on an inverse association. ANA positivity for anti-dsDNA/Sm/RNP/chromatin (cluster one) separated from anti-SSA/SSB positivity (cluster two), suggesting that these clusters relate differently to lymphocyte subset numbers. IFNα protein levels associated with cluster two, while SLEDAI-2 K projected close to anti-dsDNA in cluster one. Antiphospholipid antibody positivity (anti-CL/β2GPI, cluster three) separated from the other variables on the bottom right side of the plot.

### Lower CD4 + T cell and B cell counts in pregnant women with SLE positive for disease-specific anti-dsDNA and anti-Sm

**Fig. 4:**
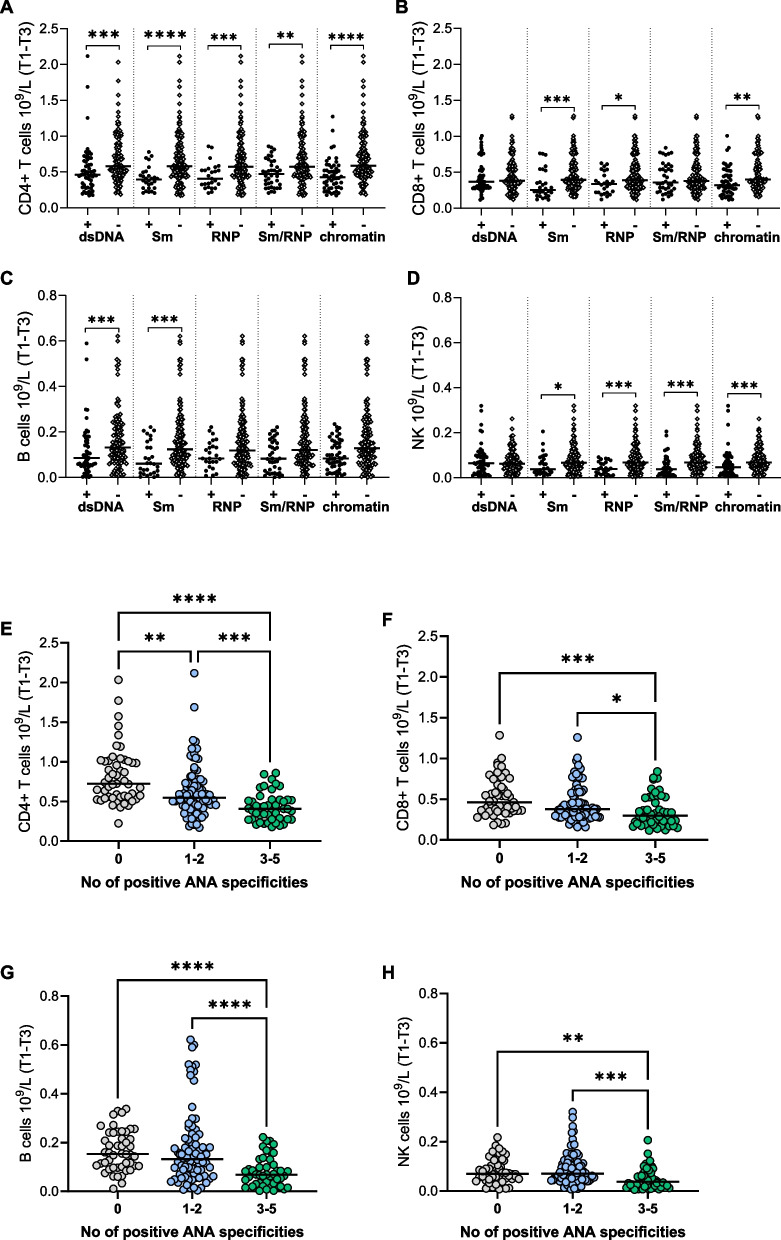
Low lymphocyte subset numbers relate to a specific cluster of ANA positivity. **(A)** CD4 + T cell, **(B)** CD8 + T cell, **(C)** B cell and **(D)** NK cell counts in women positive for anti-dsDNA, anti-Sm, anti-Sm/RNP, anti-RNP and anti-chromatin antibodies compared to women negative for respective antibody. **(E)** CD4 + T cell, **(F)** CD8 + T cell, **(G)** B cell and **(H)** NK cell counts in women negative to ANA, positive to one to two ANA or positive to three to five ANA. *p < 0.05, **p < 0.01, ***p < 0.001, ****p < 0.0001 **(A-D)** Mann–Whitney U test, **(E–H)** Kruskal–Wallis followed by Dunn’s multiple comparison test

Guided by the PCA analysis, we first investigated the association between lymphocyte subset counts and the two ANA clusters. As shown in Fig. 4A, CD4 + T cell counts were significantly lower in women positive to all ANA specificities in cluster one compared to those who were negative to respective ANA specificity. CD8 + T cell count was lower among women positive for anti-Sm, anti-RNP and anti-chromatin compared to those who were negative (Fig. 4B). B cell numbers were significantly lower in women positive for disease specific anti-dsDNA and anti-Sm compared to those who were negative (Fig. 4C). NK cell numbers were lower in women positive for all ANA in cluster one, except for anti-dsDNA, compared to those who were negative (Fig. 4D). Lymphocyte subset counts did not differ in women positive for anti-SSA or anti-SSA/SSB compared to those who were negative (Supplementary Fig. 5A-D), and none of the women were anti-SSB + /SSA-. Additionally, all lymphocyte subset counts were lower in women positive for three to five ANA compared to those who had one to two ANA and to those who were ANA negative (Fig. 4E-H). The CD4 + T cell counts were also lower in women who had one to two ANA compared to those who were negative (Fig. 4E). Few women were positive for aPL during pregnancy (anti-CL *n* = 6 and anti-β2GPI *n* = 7), and there were no significant differences in lymphocyte subset counts in women who were aPL-positive compared to negative except for NK cells counts that were higher in women with compared to without aPL (Supplementary Fig. 5E-H). In summary, lymphocyte subset counts are lower in women positive compared to negative for anti-dsDNA, anti-Sm, RNP, Sm/RNP and chromatin, while low counts are unrelated to positivity for anti-SSA/SSB and aPL in SLE pregnancies.

### Lower CD4 + T cell and B cell counts in SLE pregnancies with moderate/high disease activity

**Fig. 5:**
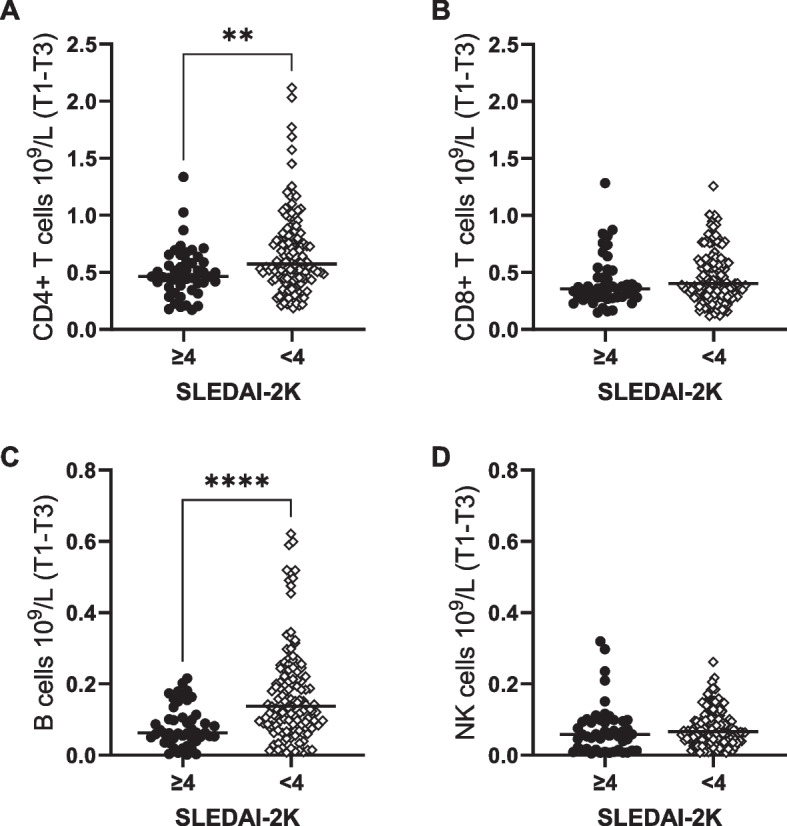
Lower CD4 + T cell and B cell numbers in pregnant women with moderate/high disease activity. **(A)** CD4 + T cell, **(B)** CD8 + T cell, **(C)** B cell and **(D)** NK cell numbers in women with moderate/high disease activity (SLEDAI-2 K ≥ 4) compared to women with no/low disease activity. **p < 0.01, ***p < 0.001, Mann–Whitney U test

Next, we examined the association between lymphocyte subsets counts and disease activity in SLE pregnancies. Both CD4 + T cell and B cell counts were lower in women with moderate/high disease activity (SLEDAI-2 K ≥ 4) compared to those with no/low disease activity, while there was no difference in numbers of CD8 + T cells and NK cells between the two groups (Fig. 5A-D). Similar results were obtained when lymphocyte subset counts for each trimester were analyzed in relation to SLEDAI-2 K for the respective trimester (Supplementary Fig. 6A-C). As expected, SLEDAI-2 K projected close to anti-dsDNA positivity in the PCA analysis, and disease activity was higher in women positive for anti-dsDNA compared to those who were negative (Supplementary Fig. 7A). SLEDAI-2 K was unrelated to other ANA fine specificities in cluster one and to number of positive ANA (Supplementary Fig. 7B-F). We have previously reported that higher proportions of low-density granulocytes in late SLE pregnancy correlate to lower gestational age at birth [24], but lymphocyte subset counts were unrelated to pregnancy duration and to giving birth to an SGA infant in women with SLE (Supplementary Fig. 8A-B and 9A-D). To conclude, pregnant women with SLE who have a moderate/high disease activity have lower circulating numbers of CD4 + T cells and B cells compared to women with no/low disease activity, but low lymphocyte subset counts do not predict shorter pregnancy duration in SLE.

### Lower CD4 + T cell counts are related to higher IFNα protein levels in SLE pregnancies

**Fig. 6:**
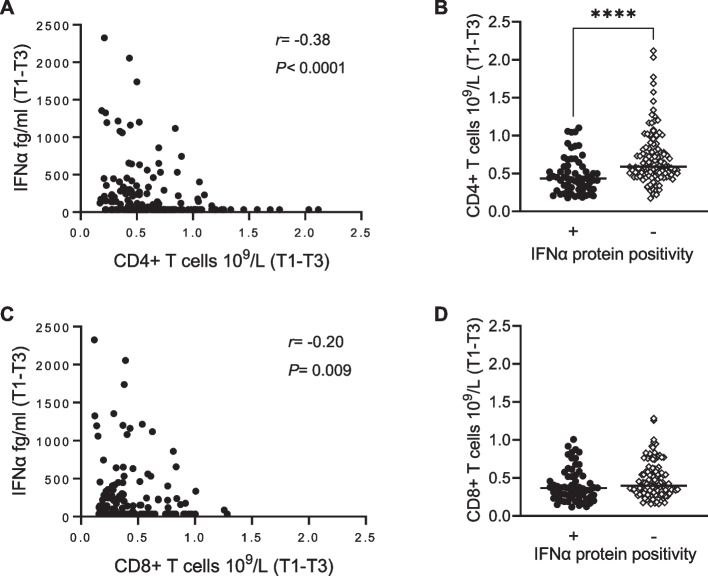
Lower CD4 + T cell counts in IFNα-positive pregnant women with SLE. **(A)** Correlation analysis of CD4 + T cell counts and IFNα protein concentrations. **(B)** Comparison of CD4 + T cell counts in pregnant women with or without IFNα protein positivity (≥ 136 fg/ml). **(C)** Correlation analysis of CD8 + T cell counts and IFNα protein concentrations. **(D)** Comparison of CD8 + T cell counts in pregnant women with or without IFNα protein positivity. ****p < 0.0001, **(A and C)** Spearman rank correlation test, **(B)** Mann–Whitney U test

In PCA, there was an inverse association between lymphocyte subset numbers and IFNα protein levels. In univariate analysis, CD4 + T cell counts inversely correlated with IFNα protein levels during SLE pregnancy (Fig. 6A), and CD4 + T cell counts were significantly lower in IFNα protein-positive compared to negative women (Fig. 6B). Numbers of CD8 + T cells showed a weaker negative correlation to IFNα protein levels and there was no difference in CD8 + T cell count between IFNα protein-positive compared to negative women (Fig. 6C-D). B cell and NK cell counts were unrelated to IFNα protein levels (Supplementary Fig. 10A-B). As previously demonstrated by others [31–33], IFNα protein levels related to number of positive ANA specificities (Supplementary Fig. 10C). Thus, pregnant women with SLE who are IFNα-positive present with lower numbers of CD4 + T cells in blood compared to those who are IFNα-negative.

## Discussion

To our knowledge, this is the first study to investigate if pregnancy affects blood lymphocyte subset counts in SLE, and if T cell, B cell and NK cell counts associate to disease-related autoantibody positivity and/or to IFNα protein concentrations during SLE pregnancy. In this longitudinal study we confirm that low total lymphocyte count is evident throughout pregnancy in women with SLE compared to HC, a well-known feature in non-pregnant patients relative to controls [34]. We also show that none of the lymphocyte subset numbers were affected by pregnancy in SLE. This contrasts with healthy pregnant women who have lower numbers of lymphocytes during pregnancy compared to the postpartum period [35, 36]. The explanation for this discrepancy could only be speculated upon, but disease-related homing of activated lymphocytes from the periphery to inflamed tissues and organs and the presence of autoantibodies with lymphocytotoxic activity may result in low lymphocyte counts that are not further affected by pregnancy in SLE [37, 38]. Although the activation status of the different lymphocyte subsets was not examined here, we have previously reported that pregnancy in SLE results in increased activation of circulating granulocytes compared to the late postpartum period [24].

Although SLE is a heterogenic disease with a variety in laboratory abnormalities and clinical features, recent detailed serological profiling has identified sets of disease-related autoantibodies that commonly occur together [16, 17, 39]. In accordance we here show for the first time in SLE pregnancy that autoantibody positivity also separated into three clusters, the first dominated by positivity for anti-dsDNA/anti-Sm/anti-RNP/anti-chromatin, the second by anti-SSA/anti-SSB and the third by anti-CL/anti-β_2_GPI. Another novel finding was that low numbers of particularly CD4 + T cells, but also B cells, CD8 + T cells and NK cells, relate to positivity for ANA specificities in cluster one, but not ANA positivity in cluster two or aPL positivity in cluster three. Whether specific lymphocyte subset counts relate to autoantibody positivity profiles has not been examined in non-pregnant patients with SLE. The use of antibody clustering to separate patients with SLE into endotypes may help to predict disease course and prognosis as patients with distinct autoantibody profiles differ with regards to immunological variables, clinical manifestations, treatment, organ involvement and long-term disease activity [16–18, 40].

Lymphopenia is associated with SLE-specific anti-dsDNA positivity, SLE-related autoantibody positivity in general, and more severe/progressive disease in non-pregnant subjects with SLE [9, 17, 18]. When we here divided total lymphocytes into subsets, only CD4 + T cell and B cell counts were lower in pregnant women positive to anti-dsDNA compared to those who were negative and counts of these subsets were also lower in women with moderate/high (SLEDAI-2 K ≥ 4) compared to no/low disease activity. Accordingly, anti-dsDNA positivity, being part of the SLEDAI-2 K score [28], was also related to higher disease activity. Anti-Sm is another SLE-specific autoantibody, but its pathologic significance is uncertain and there are conflicting data regarding its association to disease activity and clinical manifestations including lymphopenia [41–43]. We found that all lymphocyte subset counts were lower in pregnant women who were positive for anti-Sm relative to those who were negative, but anti-Sm positivity was unrelated to disease activity in our cohort. A higher number of ANA fine specificities also related to lower numbers of lymphocyte subset counts, but not to disease activity. Still, a higher number of ANA specificities could indicate a more immunologically active disease that leads to lymphocyte homing to inflamed tissue and organs, which is not captured by the SLEDAI-2 K index. Whether there is a causal relationship between low lymphocyte subsets counts and specific ANA positivity is not answered by the present data, but we add novel knowledge on how numbers of specific lymphocyte subsets in blood differ in relation to antibody positivity profiles and disease activity in SLE pregnancies.

Lower total lymphocyte counts have also been reported in IFNα positive compared to negative non-pregnant patients with SLE [30]. We found that specifically CD4 + T cell counts, but not counts of any other lymphocyte subset, were lower in IFNα-positive pregnant women with SLE compared to those who were negative. Our finding is in line with recent scRNA-seq data showing that a reduction of naïve CD4 + T cells correlates with increased expression of type I IFN regulated genes in monocytes in non-pregnant individuals with SLE [21]. Whether there is a direct effect of IFNα on CD4 + T cell numbers in blood is unclear, but administration of IFNα in healthy volunteers leads to a drastic decrease of total lymphocyte numbers in blood [44, 45]. Mouse models suggest a partial mechanistic explanation for this phenomenon by inhibited egress of CD4 + T cells, CD8 + T cells and CD19 + B cells from lymph nodes, as treatment with the IFNα inducer poly (I:C) retains lymphocytes in lymph nodes via regulation of CD69 and sphingosine 1-phosphate receptor-1 (S1P1) expression [46]. Still, the relationship between IFNα and low CD4 + T cell counts remains to be examined further.

Medication may affect numbers of lymphocyte subsets in blood. Indeed, pregnant women with SLE who were treated with azathioprine had lower numbers of NK cells and B cells compared to women who were not treated. In accordance with this, azathioprine use is related to reduced numbers and proportions of NK cells and B cells in non-pregnant subjects with SLE, inflammatory bowel disease or ANCA-associated vasculitis [21, 47–50]. A proposed mechanism for azathioprine-related decrease in NK cells is caspase 3- and 9-induced apoptosis [49]. Additionally, we also found lower B cell counts in women treated with prednisone or heparin compared to those who were not. For prednisone, similar results were reported from a small cohort of patients with different autoimmune disorders [51], and in a small cohort of healthy volunteers [52]. Importantly, we also found a treatment-independent decrease in both NK cells and B cells in SLE compared to HC pregnancies.

A strength of the study is the inclusion of well-characterized patients and controls from whom samples have been prospectively collected in parallel during pregnancy and late postpartum. Others are that all flow cytometry analyses were performed on fresh blood in one laboratory on the same instrument and cross-sectional analysis of autoantibody positivity was analyzed with well-standardized methods by staff in an accredited hospital laboratory. Our study also has limitations. The cohort included few women with moderate or high SLE disease activity and therefore our results reflect a well-controlled cohort of pregnant women with SLE. Moreover, the study includes missing data, mainly due to missing blood samples from the first trimester, among pregnant women with SLE.

To conclude, we report that pregnancy in women with SLE has no effect on blood lymphocyte subset counts but SLE pregnancies are featured by a treatment-independent decrease in blood lymphocyte counts compared to healthy pregnant women. Moreover, low counts of specific lymphocyte subsets relate differentially to disease-related autoantibody positivity, IFNα protein levels and disease activity in SLE pregnancy. Still, further studies are needed to decipher the immunological characteristics of SLE phenotypes based of antibody profiles in more detail, and to investigate if specific subgroups are related to an increased risk for pregnancy complications.

### Supplementary Information

**Supplementary Material 1. **

**Supplementary Material 2. **

## Abbreviations

ACR: American college of rheumatology
ANA: Antinuclear antibodies
aPL: Antiphospholipid andibodies
CL: Cardiolipin
dsDNA: Double-stranded DNA
ELISA: Enzyme-linked immunosorbent assay
HC: Healthy controls
IFN: Interferon
LAC: Lupus anticoagulant
OPLS: Orthogonal partial least square
PCA: Principal component analysis
RNP: Ribonucleoprotein
Simoa: Single molecule array
SLE: Systemic lupus erythematosus
SLEDAI-2 K: SLE disease activity index 2000
SLICC: Systemic lupus international collaborating clinics
Sm: Smith
SSA: Sjögren’s syndrome antigen A
SSB: Sjögren’s syndrome antigen B
β_2_GPI: β_2_ Glycoprotein I
