# Supplementary material for: Low CD4 + T cell count is related to specific anti-nuclear antibodies, IFNα protein positivity and disease activity in systemic lupus erythematosus pregnancy
Source: Arthritis Res Ther. 2024 Mar 9;26:65. doi: 10.1186/s13075-024-03301-0 (PMC10924387; doi:10.1186/s13075-024-03301-0)
Supplement: Supplementary file 1 — Supplementary Material 1. [file 13075_2024_3301_MOESM1_ESM.pptx]

## Slide 1
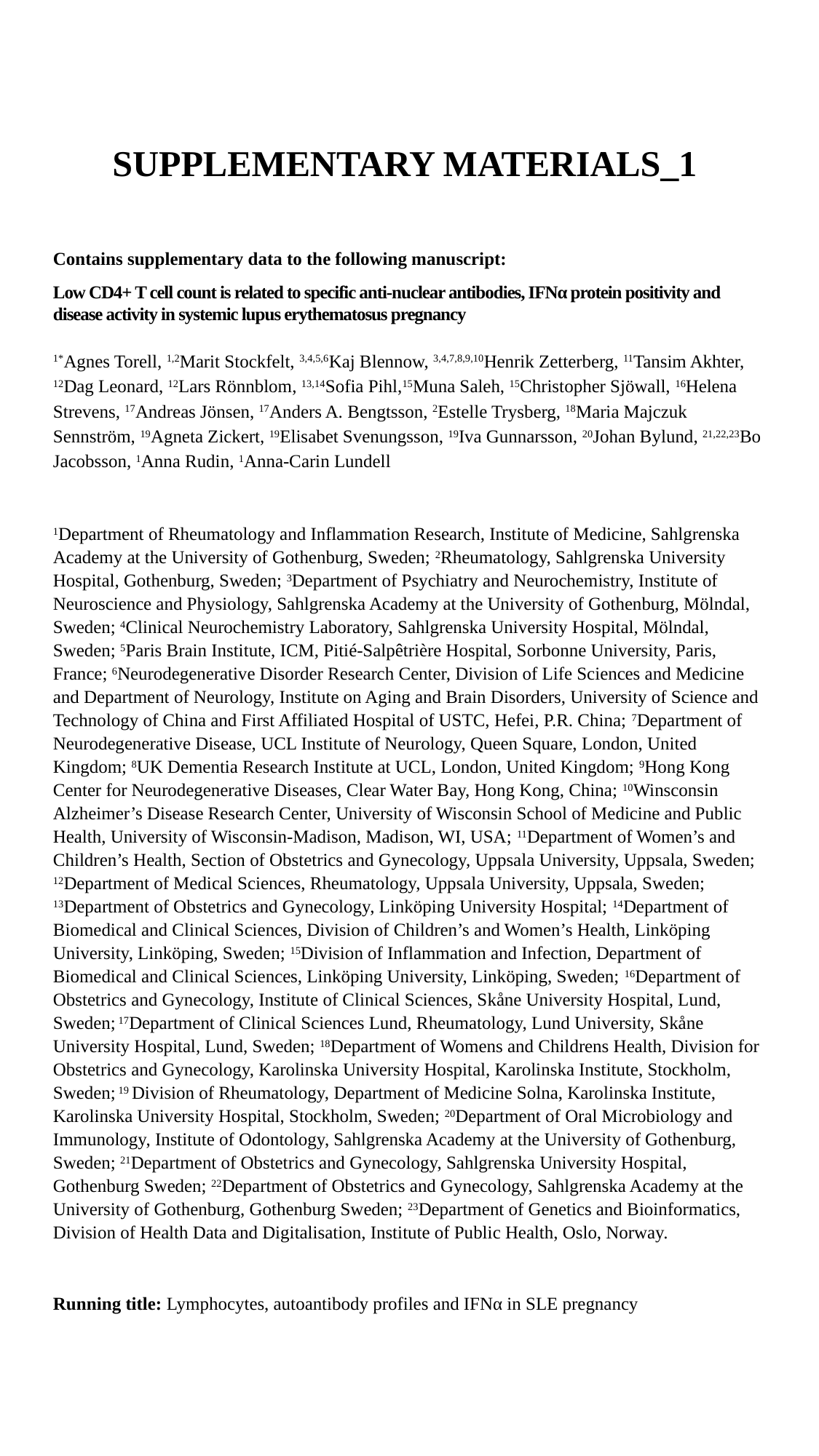

SUPPLEMENTARY MATERIALS_1
Contains supplementary data to the following manuscript:
Low CD4+ T cell count is related to specific anti-nuclear antibodies, IFNα protein positivity and disease activity in systemic lupus erythematosus pregnancy
1*Agnes Torell, 1,2Marit Stockfelt, 3,4,5,6Kaj Blennow, 3,4,7,8,9,10Henrik Zetterberg, 11Tansim Akhter, 12Dag Leonard, 12Lars Rönnblom, 13,14Sofia Pihl,15Muna Saleh, 15Christopher Sjöwall, 16Helena Strevens, 17Andreas Jönsen, 17Anders A. Bengtsson, 2Estelle Trysberg, 18Maria Majczuk Sennström, 19Agneta Zickert, 19Elisabet Svenungsson, 19Iva Gunnarsson, 20Johan Bylund, 21,22,23Bo Jacobsson, 1Anna Rudin, 1Anna-Carin Lundell
1Department of Rheumatology and Inflammation Research, Institute of Medicine, Sahlgrenska Academy at the University of Gothenburg, Sweden; 2Rheumatology, Sahlgrenska University Hospital, Gothenburg, Sweden; 3Department of Psychiatry and Neurochemistry, Institute of Neuroscience and Physiology, Sahlgrenska Academy at the University of Gothenburg, Mölndal, Sweden; 4Clinical Neurochemistry Laboratory, Sahlgrenska University Hospital, Mölndal, Sweden; 5Paris Brain Institute, ICM, Pitié-Salpêtrière Hospital, Sorbonne University, Paris, France; 6Neurodegenerative Disorder Research Center, Division of Life Sciences and Medicine and Department of Neurology, Institute on Aging and Brain Disorders, University of Science and Technology of China and First Affiliated Hospital of USTC, Hefei, P.R. China; 7Department of Neurodegenerative Disease, UCL Institute of Neurology, Queen Square, London, United Kingdom; 8UK Dementia Research Institute at UCL, London, United Kingdom; 9Hong Kong Center for Neurodegenerative Diseases, Clear Water Bay, Hong Kong, China; 10Winsconsin Alzheimer’s Disease Research Center, University of Wisconsin School of Medicine and Public Health, University of Wisconsin-Madison, Madison, WI, USA; 11Department of Women’s and Children’s Health, Section of Obstetrics and Gynecology, Uppsala University, Uppsala, Sweden; 12Department of Medical Sciences, Rheumatology, Uppsala University, Uppsala, Sweden; 13Department of Obstetrics and Gynecology, Linköping University Hospital; 14Department of Biomedical and Clinical Sciences, Division of Children’s and Women’s Health, Linköping University, Linköping, Sweden; 15Division of Inflammation and Infection, Department of Biomedical and Clinical Sciences, Linköping University, Linköping, Sweden; 16Department of Obstetrics and Gynecology, Institute of Clinical Sciences, Skåne University Hospital, Lund, Sweden; 17Department of Clinical Sciences Lund, Rheumatology, Lund University, Skåne University Hospital, Lund, Sweden; 18Department of Womens and Childrens Health, Division for Obstetrics and Gynecology, Karolinska University Hospital, Karolinska Institute, Stockholm, Sweden; 19 Division of Rheumatology, Department of Medicine Solna, Karolinska Institute, Karolinska University Hospital, Stockholm, Sweden; 20Department of Oral Microbiology and Immunology, Institute of Odontology, Sahlgrenska Academy at the University of Gothenburg, Sweden; 21Department of Obstetrics and Gynecology, Sahlgrenska University Hospital, Gothenburg Sweden; 22Department of Obstetrics and Gynecology, Sahlgrenska Academy at the University of Gothenburg, Gothenburg Sweden; 23Department of Genetics and Bioinformatics, Division of Health Data and Digitalisation, Institute of Public Health, Oslo, Norway.
Running title: Lymphocytes, autoantibody profiles and IFNα in SLE pregnancy

## Slide 2
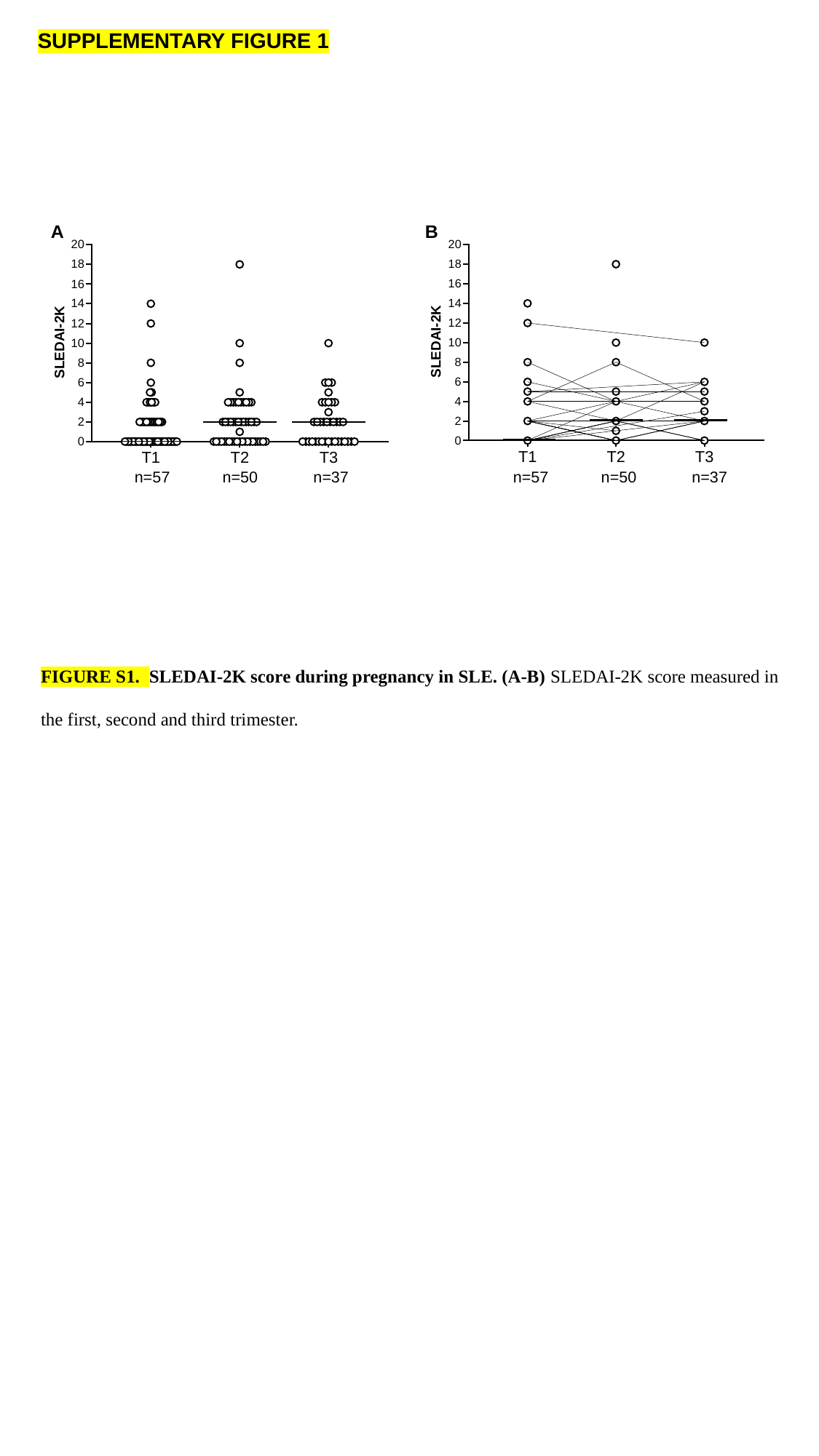

SUPPLEMENTARY FIGURE 1
A
B
FIGURE S1. SLEDAI-2K score during pregnancy in SLE. (A-B) SLEDAI-2K score measured in the first, second and third trimester.

## Slide 3
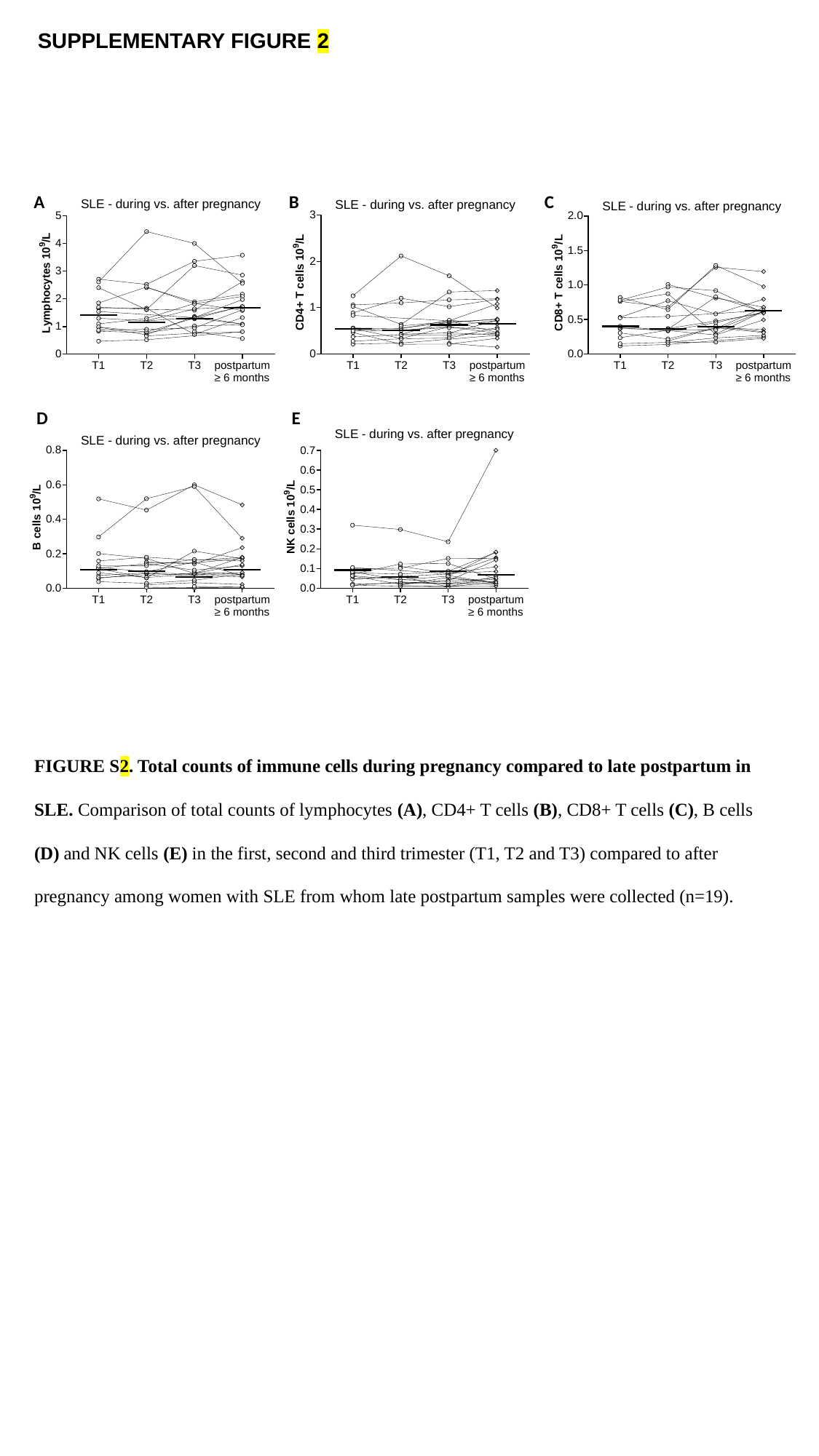

SUPPLEMENTARY FIGURE 2
C
B
A
E
D
FIGURE S2. Total counts of immune cells during pregnancy compared to late postpartum in SLE. Comparison of total counts of lymphocytes (A), CD4+ T cells (B), CD8+ T cells (C), B cells (D) and NK cells (E) in the first, second and third trimester (T1, T2 and T3) compared to after pregnancy among women with SLE from whom late postpartum samples were collected (n=19).

## Slide 4
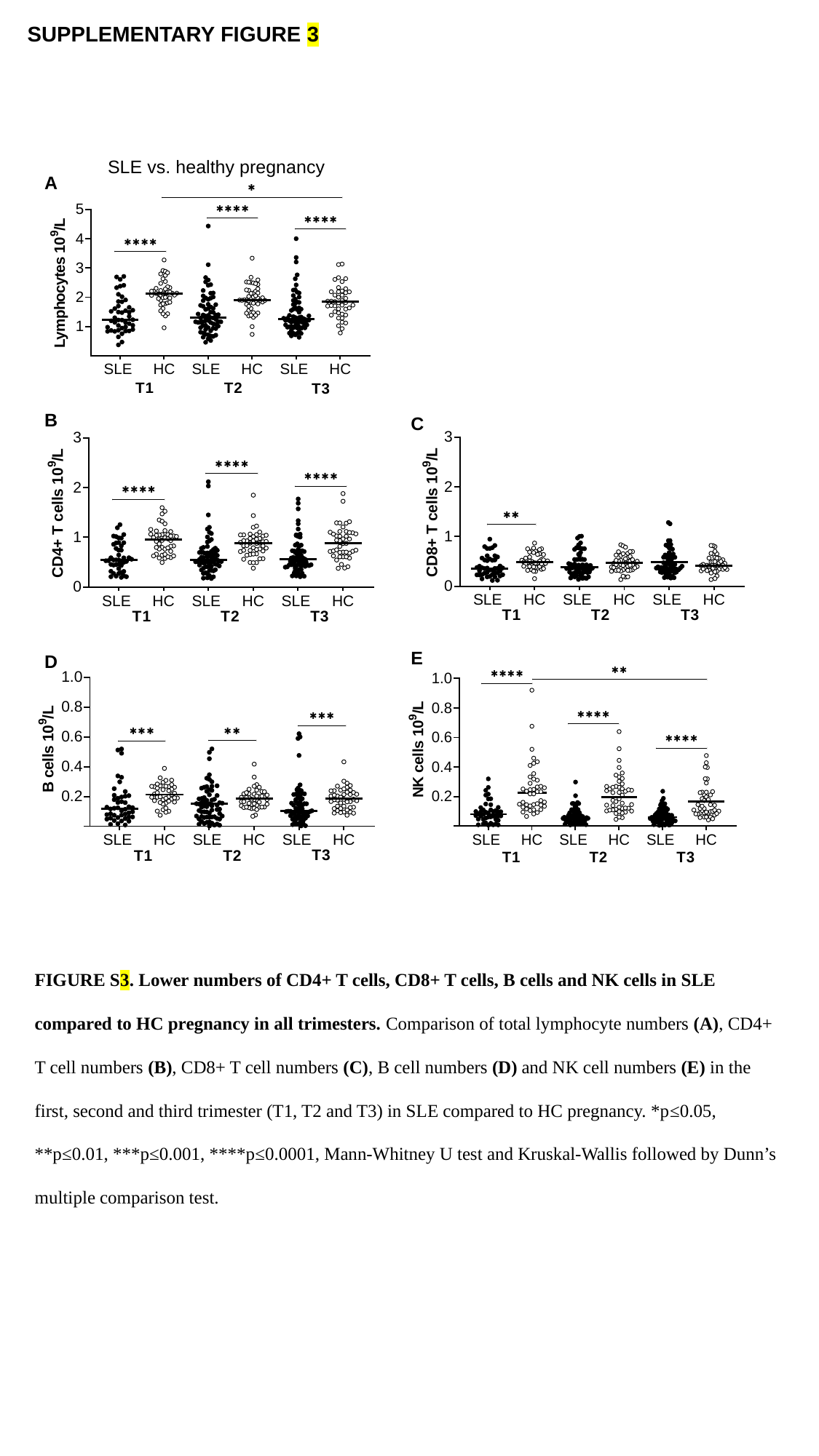

SUPPLEMENTARY FIGURE 3
SLE vs. healthy pregnancy
A
B
C
E
D
FIGURE S3. Lower numbers of CD4+ T cells, CD8+ T cells, B cells and NK cells in SLE compared to HC pregnancy in all trimesters. Comparison of total lymphocyte numbers (A), CD4+ T cell numbers (B), CD8+ T cell numbers (C), B cell numbers (D) and NK cell numbers (E) in the first, second and third trimester (T1, T2 and T3) in SLE compared to HC pregnancy. *p≤0.05, **p≤0.01, ***p≤0.001, ****p≤0.0001, Mann-Whitney U test and Kruskal-Wallis followed by Dunn’s multiple comparison test.

## Slide 5
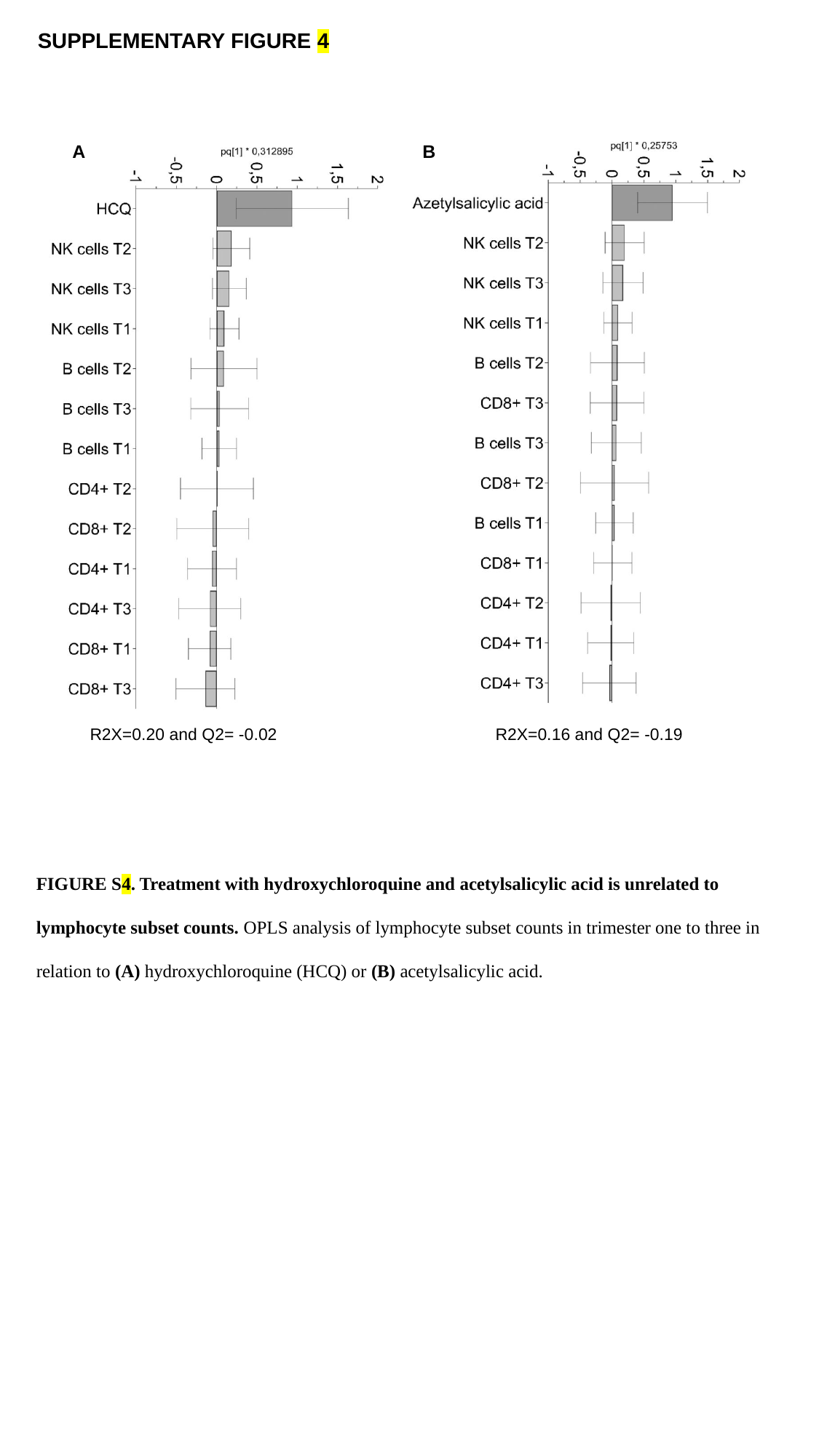

SUPPLEMENTARY FIGURE 4
A
B
R2X=0.20 and Q2= -0.02
R2X=0.16 and Q2= -0.19
FIGURE S4. Treatment with hydroxychloroquine and acetylsalicylic acid is unrelated to lymphocyte subset counts. OPLS analysis of lymphocyte subset counts in trimester one to three in relation to (A) hydroxychloroquine (HCQ) or (B) acetylsalicylic acid.

## Slide 6
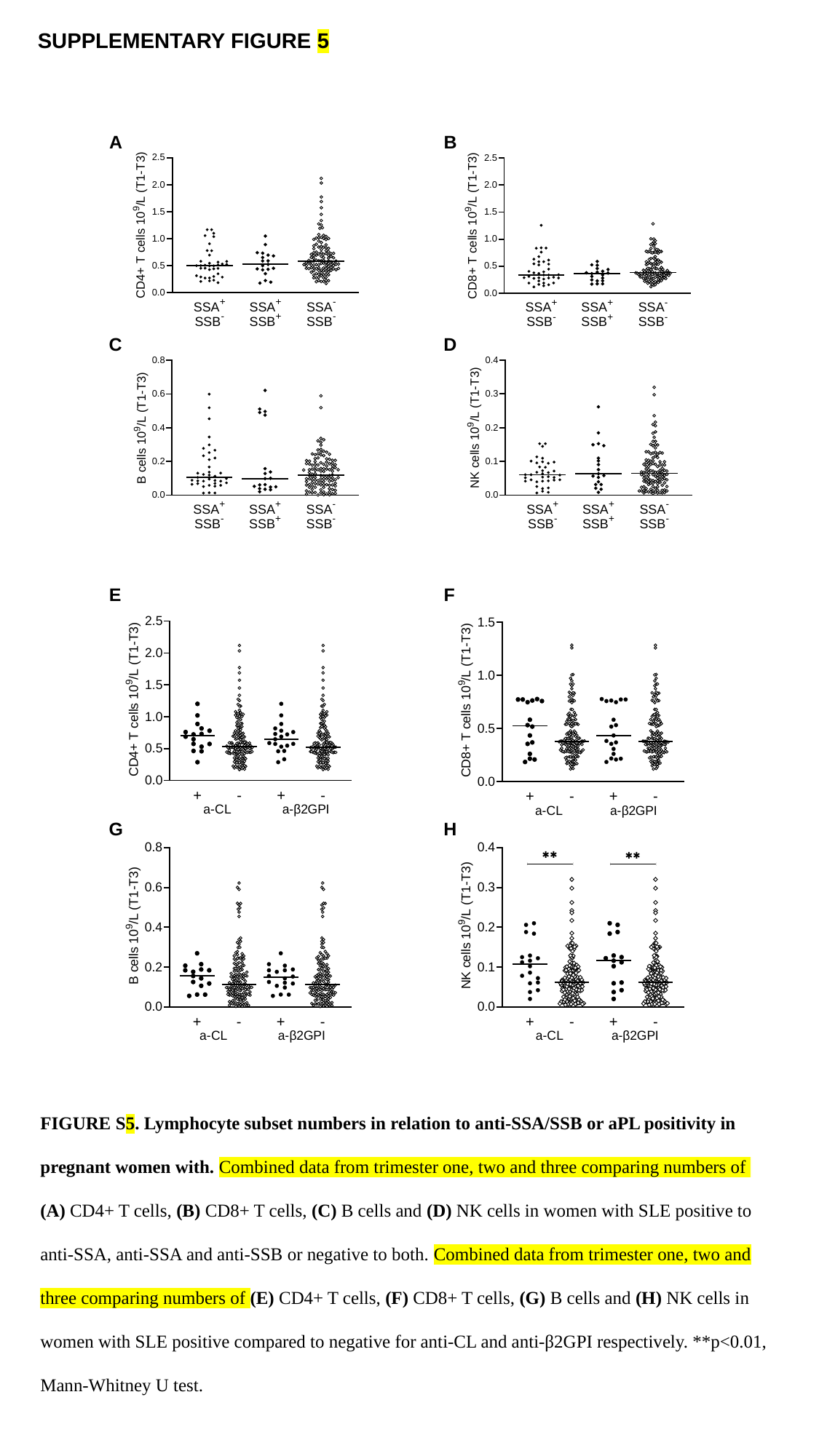

SUPPLEMENTARY FIGURE 5
A
B
C
D
E
F
G
H
FIGURE S5. Lymphocyte subset numbers in relation to anti-SSA/SSB or aPL positivity in pregnant women with. Combined data from trimester one, two and three comparing numbers of (A) CD4+ T cells, (B) CD8+ T cells, (C) B cells and (D) NK cells in women with SLE positive to anti-SSA, anti-SSA and anti-SSB or negative to both. Combined data from trimester one, two and three comparing numbers of (E) CD4+ T cells, (F) CD8+ T cells, (G) B cells and (H) NK cells in women with SLE positive compared to negative for anti-CL and anti-β2GPI respectively. **p<0.01, Mann-Whitney U test.

## Slide 7
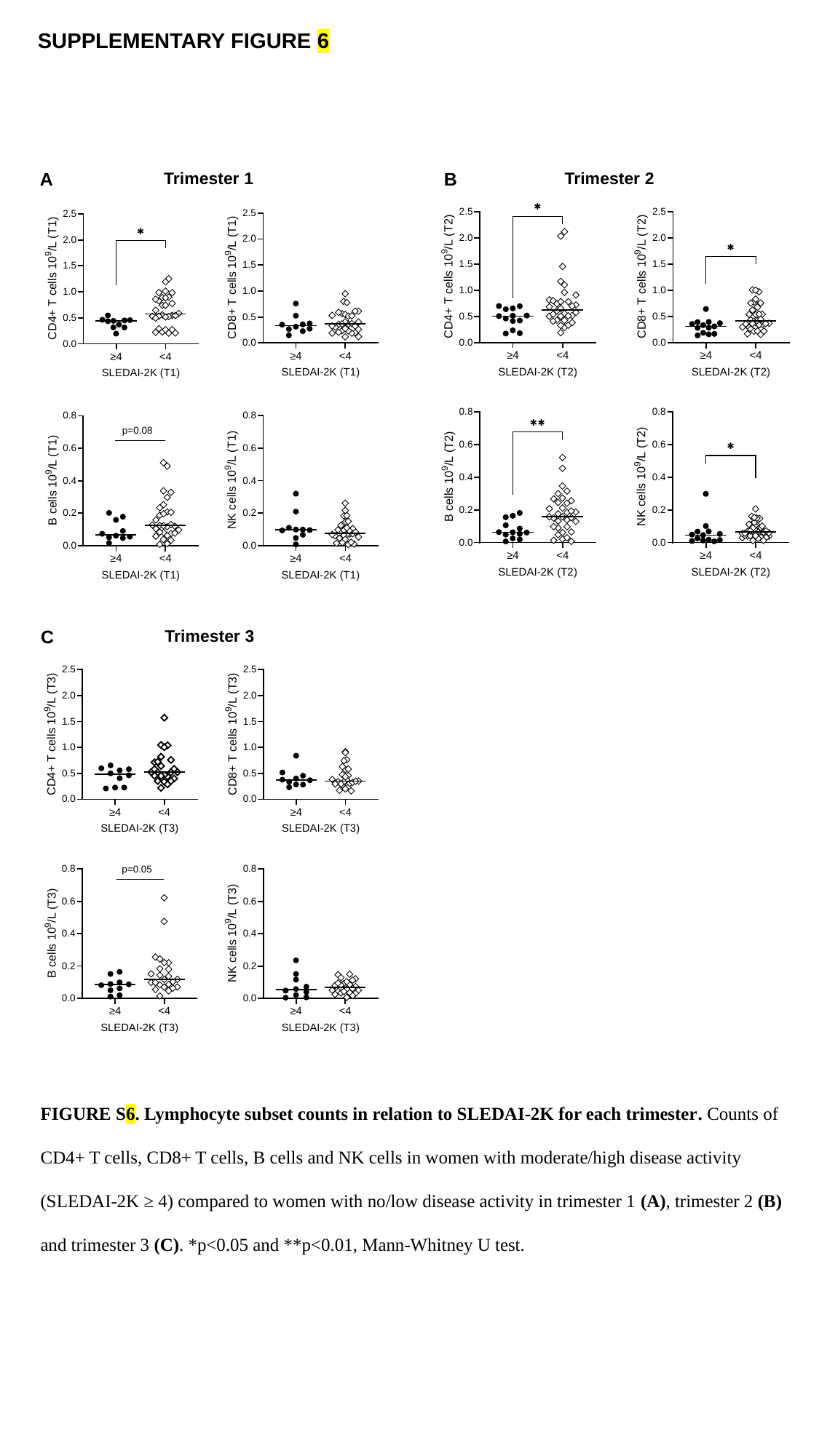

SUPPLEMENTARY FIGURE 6
A
Trimester 1
B
Trimester 2
C
Trimester 3
FIGURE S6. Lymphocyte subset counts in relation to SLEDAI-2K for each trimester. Counts of CD4+ T cells, CD8+ T cells, B cells and NK cells in women with moderate/high disease activity (SLEDAI-2K ≥ 4) compared to women with no/low disease activity in trimester 1 (A), trimester 2 (B) and trimester 3 (C). *p<0.05 and **p<0.01, Mann-Whitney U test.

## Slide 8
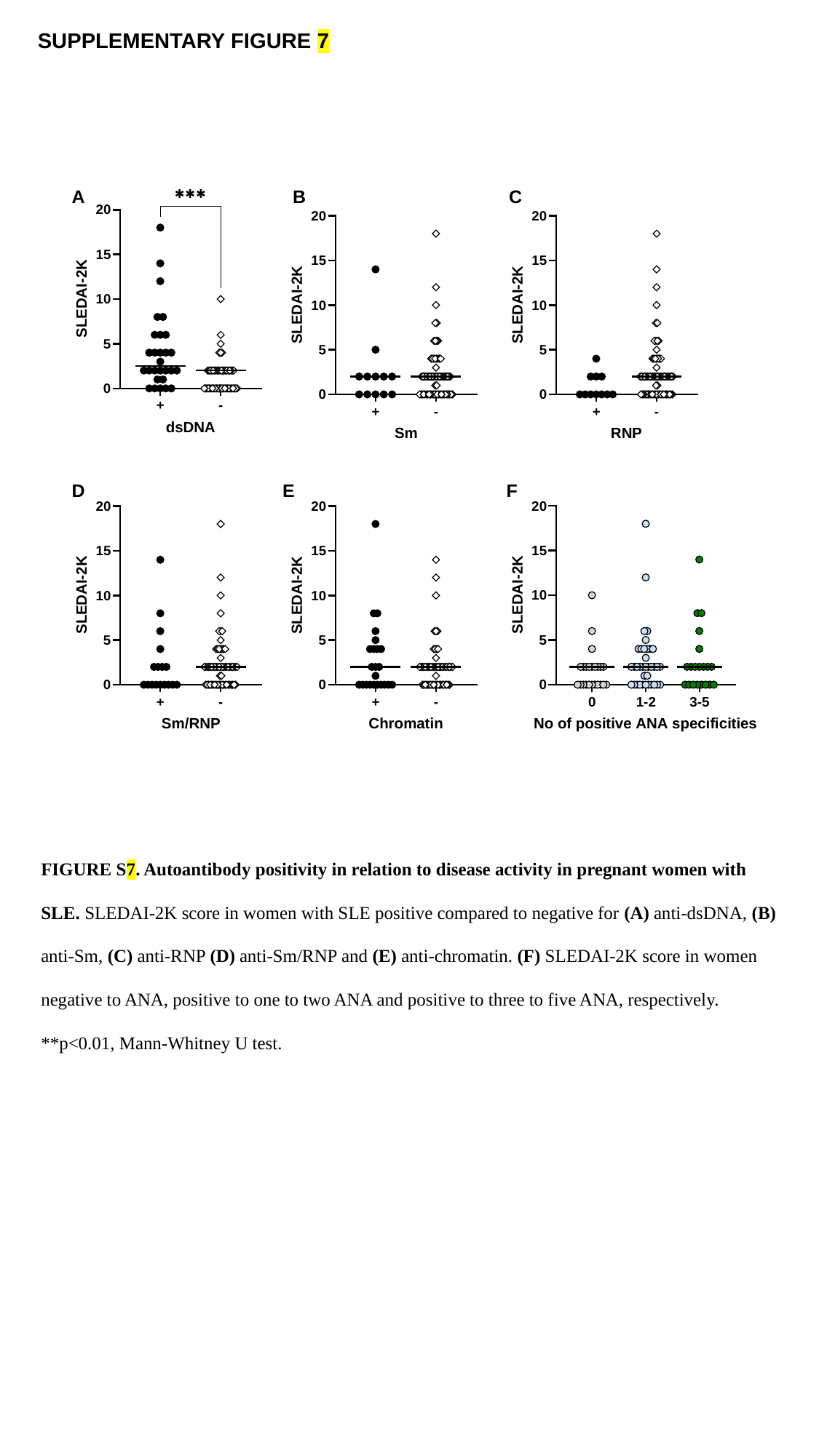

SUPPLEMENTARY FIGURE 7
A
B
C
D
E
F
FIGURE S7. Autoantibody positivity in relation to disease activity in pregnant women with SLE. SLEDAI-2K score in women with SLE positive compared to negative for (A) anti-dsDNA, (B) anti-Sm, (C) anti-RNP (D) anti-Sm/RNP and (E) anti-chromatin. (F) SLEDAI-2K score in women negative to ANA, positive to one to two ANA and positive to three to five ANA, respectively. **p<0.01, Mann-Whitney U test.

## Slide 9
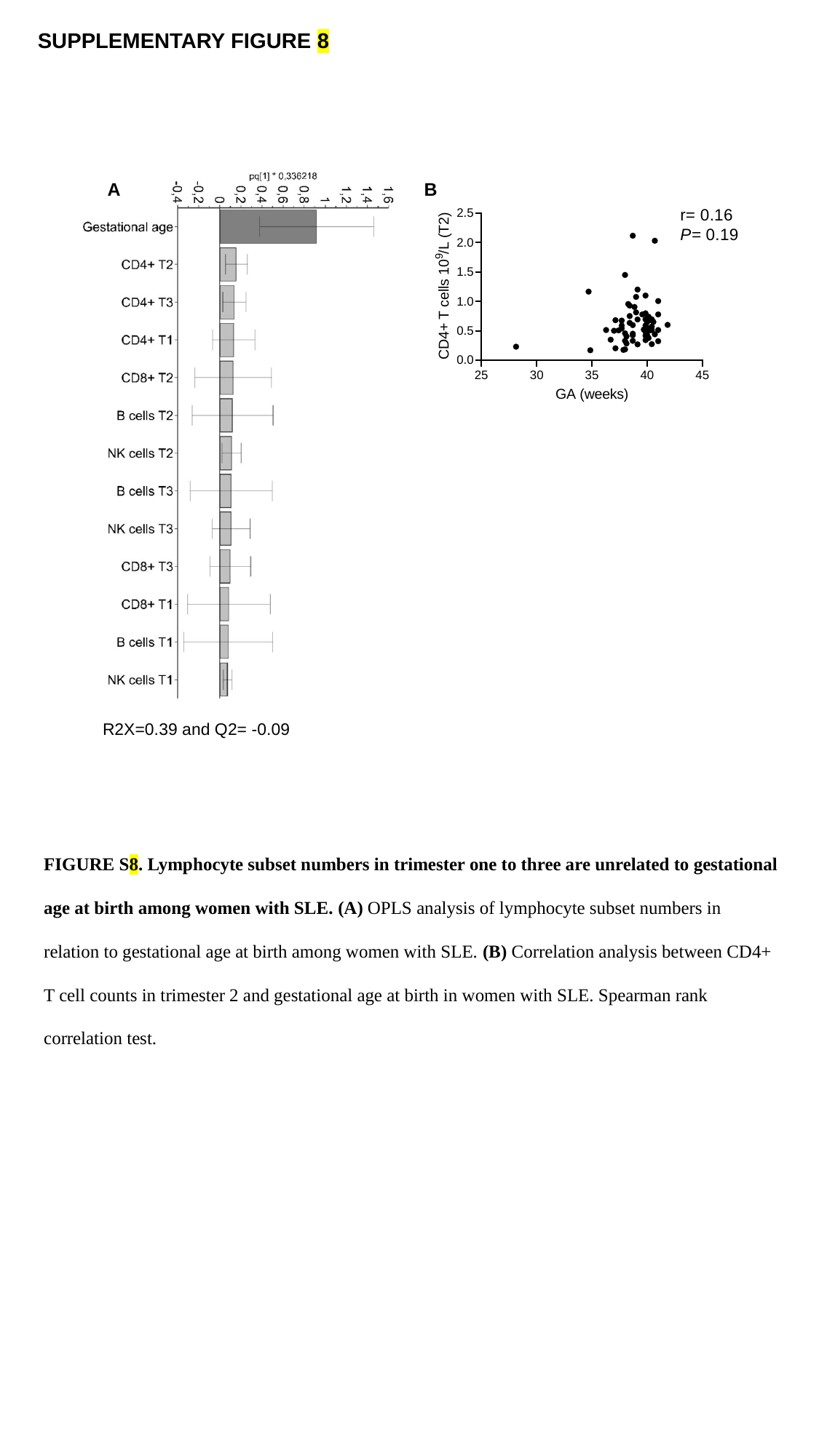

SUPPLEMENTARY FIGURE 8
B
A
R2X=0.39 and Q2= -0.09
FIGURE S8. Lymphocyte subset numbers in trimester one to three are unrelated to gestational age at birth among women with SLE. (A) OPLS analysis of lymphocyte subset numbers in relation to gestational age at birth among women with SLE. (B) Correlation analysis between CD4+ T cell counts in trimester 2 and gestational age at birth in women with SLE. Spearman rank correlation test.

## Slide 10
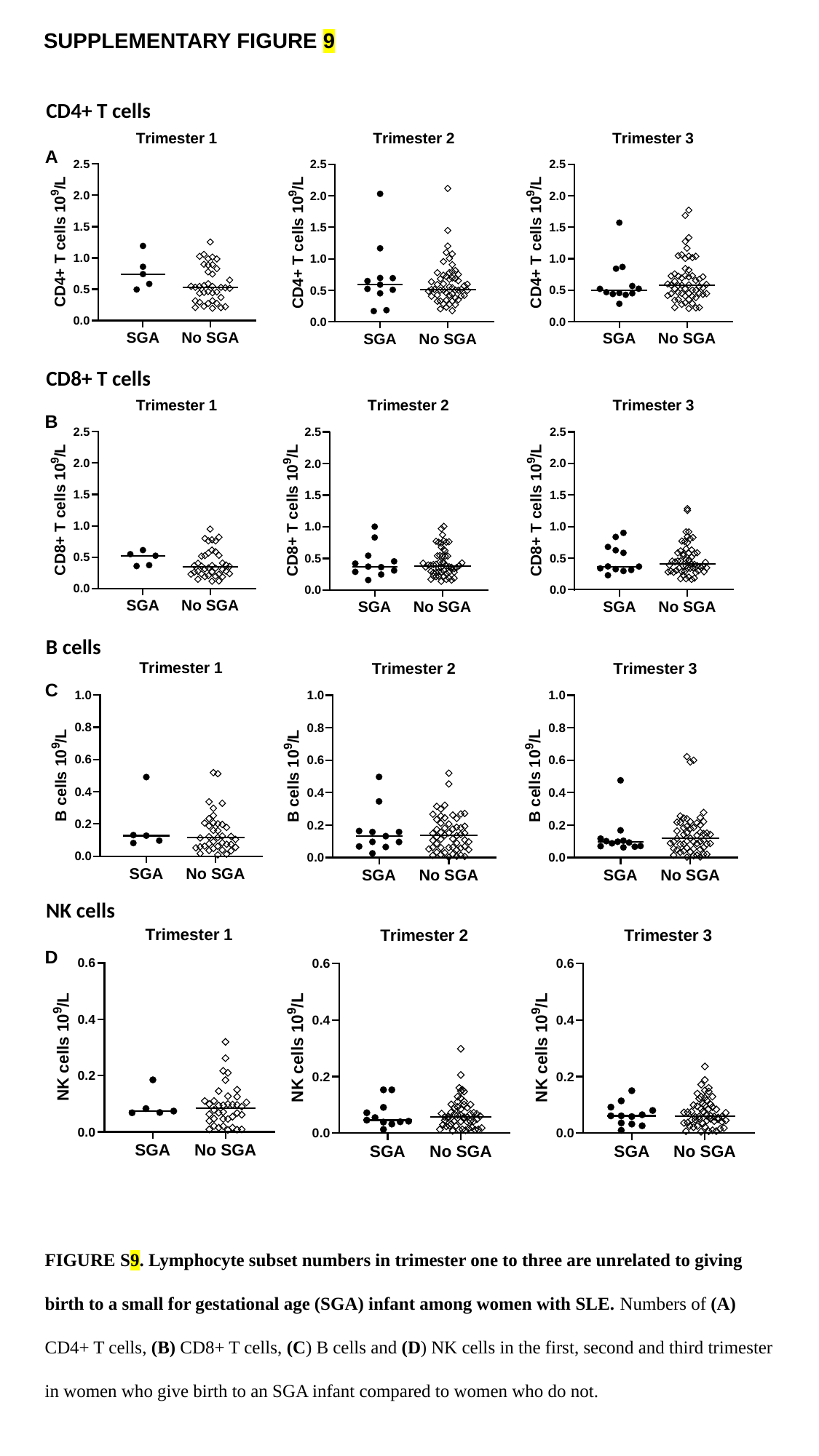

SUPPLEMENTARY FIGURE 9
CD4+ T cells
A
CD8+ T cells
B
B cells
C
NK cells
D
FIGURE S9. Lymphocyte subset numbers in trimester one to three are unrelated to giving birth to a small for gestational age (SGA) infant among women with SLE. Numbers of (A) CD4+ T cells, (B) CD8+ T cells, (C) B cells and (D) NK cells in the first, second and third trimester in women who give birth to an SGA infant compared to women who do not.

## Slide 11
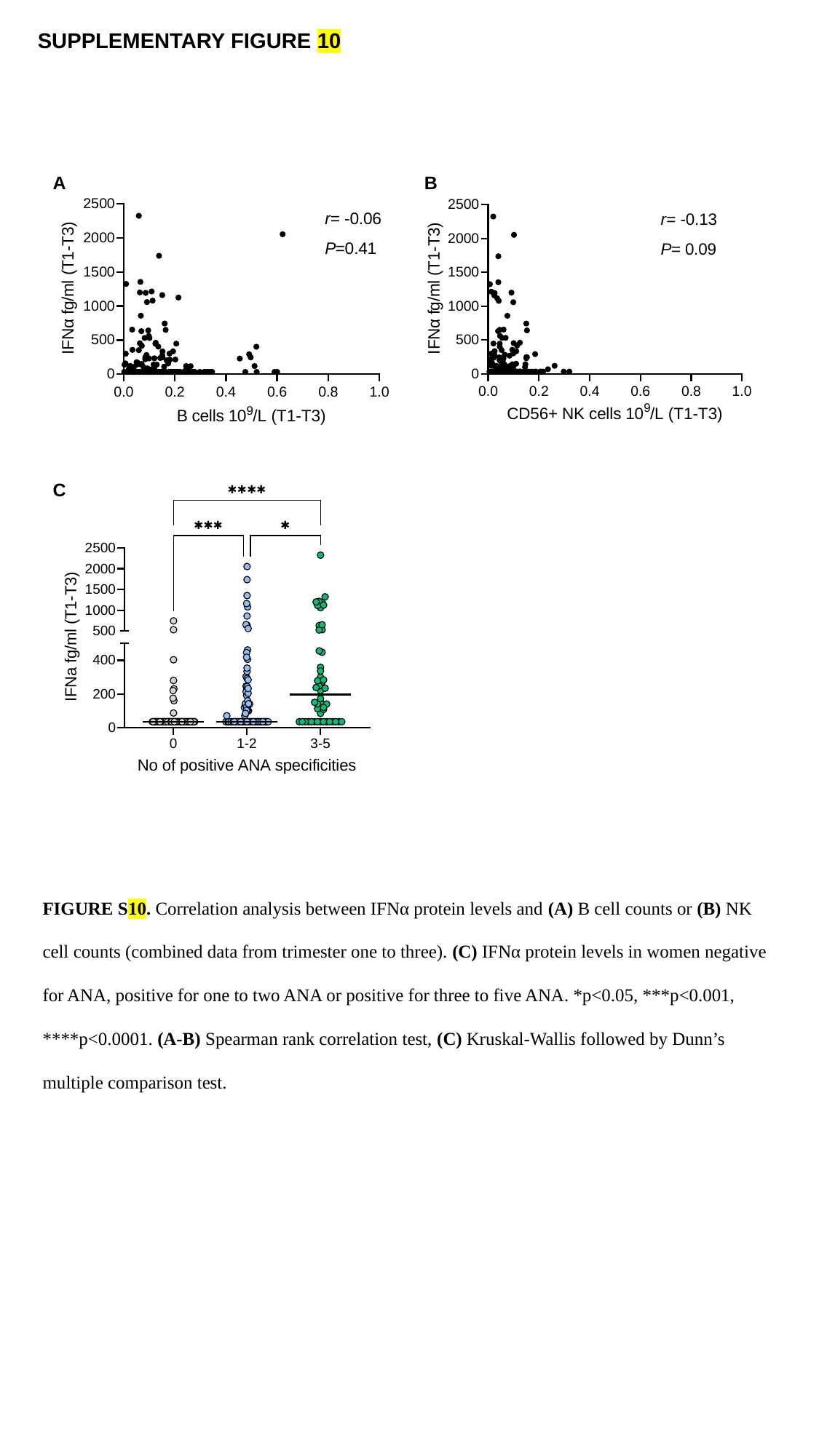

SUPPLEMENTARY FIGURE 10
A
B
C
FIGURE S10. Correlation analysis between IFNα protein levels and (A) B cell counts or (B) NK cell counts (combined data from trimester one to three). (C) IFNα protein levels in women negative for ANA, positive for one to two ANA or positive for three to five ANA. *p<0.05, ***p<0.001, ****p<0.0001. (A-B) Spearman rank correlation test, (C) Kruskal-Wallis followed by Dunn’s multiple comparison test.
