# Supplementary material for: Low CD4 + T cell count is related to specific anti-nuclear antibodies, IFNα protein positivity and disease activity in systemic lupus erythematosus pregnancy
Source: Arthritis Res Ther. 2024 Mar 9;26:65. doi: 10.1186/s13075-024-03301-0 (PMC10924387; doi:10.1186/s13075-024-03301-0)
Supplement: Supplementary file 2 — Supplementary Material 2. [file 13075_2024_3301_MOESM2_ESM.docx]

## **SUPPLEMENTARY MATERIAL_2**

**Contains supplementary data to the following manuscript:**

**Low CD4+ T cell count is related to specific anti-nuclear antibodies, IFNα protein positivity and disease activity in systemic lupus erythematosus pregnancy**

^1*^Agnes Torell, ^1,2^Marit Stockfelt, ^3,4,5,6^Kaj Blennow, ^3,4,7,8,9,10^Henrik Zetterberg, ^11^Tansim Akhter, ^12^Dag Leonard, ^12^Lars Rönnblom, ^13,14^Sofia Pihl,^15^Muna Saleh, ^15^Christopher Sjöwall, ^16^Helena Strevens, ^17^Andreas Jönsen, ^17^Anders A. Bengtsson, ^2^Estelle Trysberg, ^18^Maria Majczuk Sennström, ^19^Agneta Zickert, ^19^Elisabet Svenungsson, ^19^Iva Gunnarsson, ^20^Johan Bylund, ^21,22,23^Bo Jacobsson, ^1^Anna Rudin, ^1^Anna-Carin Lundell

^1^Department of Rheumatology and Inflammation Research, Institute of Medicine, Sahlgrenska Academy at the University of Gothenburg, Sweden; ^2^Rheumatology, Sahlgrenska University Hospital, Gothenburg, Sweden; ^3^Department of Psychiatry and Neurochemistry, Institute of Neuroscience and Physiology, Sahlgrenska Academy at the University of Gothenburg, Mölndal, Sweden; ^4^Clinical Neurochemistry Laboratory, Sahlgrenska University Hospital, Mölndal, Sweden; ^5^Paris Brain Institute, ICM, Pitié-Salpêtrière Hospital, Sorbonne University, Paris, France; ^6^Neurodegenerative Disorder Research Center, Division of Life Sciences and Medicine and Department of Neurology, Institute on Aging and Brain Disorders, University of Science and Technology of China and First Affiliated Hospital of USTC, Hefei, P.R. China; ^7^Department of Neurodegenerative Disease, UCL Institute of Neurology, Queen Square, London, United Kingdom; ^8^UK Dementia Research Institute at UCL, London, United Kingdom; ^9^Hong Kong Center for Neurodegenerative Diseases, Clear Water Bay, Hong Kong, China; ^10^Winsconsin Alzheimer’s Disease Research Center, University of Wisconsin School of Medicine and Public Health, University of Wisconsin-Madison, Madison, WI, USA; ^11^Department of Women’s and Children’s Health, Section of Obstetrics and Gynecology, Uppsala University, Uppsala, Sweden; ^12^Department of Medical Sciences, Rheumatology, Uppsala University, Uppsala, Sweden; ^13^Department of Obstetrics and Gynecology, Linköping University Hospital; ^14^Department of Biomedical and Clinical Sciences, Division of Children’s and Women’s Health, Linköping University, Linköping, Sweden; ^15^Division of Inflammation and Infection, Department of Biomedical and Clinical Sciences, Linköping University, Linköping, Sweden; ^16^Department of Obstetrics and Gynecology, Institute of Clinical Sciences, Skåne University Hospital, Lund, Sweden; ^17^Department of Clinical Sciences Lund, Rheumatology, Lund University, Skåne University Hospital, Lund, Sweden; ^18^Department of Womens and Childrens Health, Division for Obstetrics and Gynecology, Karolinska University Hospital, Karolinska Institute, Stockholm, Sweden; ^19^ Division of Rheumatology, Department of Medicine Solna, Karolinska Institute, Karolinska University Hospital, Stockholm, Sweden; ^20^Department of Oral Microbiology and Immunology, Institute of Odontology, Sahlgrenska Academy at the University of Gothenburg, Sweden; ^21^Department of Obstetrics and Gynecology, Sahlgrenska University Hospital, Gothenburg Sweden; ^22^Department of Obstetrics and Gynecology, Sahlgrenska Academy at the University of Gothenburg, Gothenburg Sweden; ^23^Department of Genetics and Bioinformatics, Division of Health Data and Digitalisation, Institute of Public Health, Oslo, Norway.

**Running title:** Lymphocytes, autoantibody profiles and IFNα in SLE pregnancy

## **Supplementary Table 1.** Number of SLEDAI-2K score assessment during pregnancy

|  | Trimester one  week 10-12 | Trimester two  week 18-20 | Trimester three  week 32-34 |
| --- | --- | --- | --- |
| SLE (n=80) | 57 | 50 | 37 |

## **Supplementary Table 2.** Number of collected blood samples

|  | Trimester one  week 10-12 | Trimester two  week 18-20 | Trimester three  week 32-34 | Late postpartum  ≥ 6 months after delivery |
| --- | --- | --- | --- | --- |
| SLE (n=80) | 44 | 66 | 70 | 19 |
| HC (n=51) | 44 | 46 | 43 |  |

## **Supplementary Table 3:** Antibodies used for flow cytometry

| Flow cytometry reactivity | Conjugate | Clone | Company |
| --- | --- | --- | --- |
| CD45 | PerCP | 2D1 | BD Biosciences |
| CD3 | FITC | UCHT1 | BD Biosciences |
| CD4 | APC-H7 | SK3 | BD Biosciences |
| CD8 | PE-Cy7 | RPA-T8 | BD Biosciences |
| CD19 | APC-H7 | SJ25C1 | BD Biosciences |
| CD20 | APC-H7 | L27 | BD Biosciences |
| CD56 | BV421 | HCD56 | Nordic Biosite |
